# Design and Assessment of Control Maps for Multi-Channel sEMG-Driven Prostheses and Supernumerary Limbs

**DOI:** 10.3389/fnbot.2019.00026

**Published:** 2019-05-29

**Authors:** Michele Maimeri, Cosimo Della Santina, Cristina Piazza, Matteo Rossi, Manuel G. Catalano, Giorgio Grioli

**Affiliations:** ^1^Soft Robotics for Human Cooperation and Rehabilitation, Istituto Italiano di Tecnologia, Genoa, Italy; ^2^Research Center “Enrico Piaggio”, University of Pisa, Pisa, Italy; ^3^Dipartimento di Ingegneria Informatica, University of Pisa, Pisa, Italy

**Keywords:** myoelectric control, upper limb prostheses, extra limbs, supernumerary limbs, soft hands, proportional and simultaneous control

## Abstract

Proportional and simultaneous control algorithms are considered as one of the most effective ways of mapping electromyographic signals to an artificial device. However, the applicability of these methods is limited by the high number of electromyographic features that they require to operate—typically twice as many the actuators to be controlled. Indeed, extracting many independent electromyographic signals is challenging for a number of reasons—ranging from technological to anatomical. On the contrary, the number of actively moving parts in classic prostheses or extra-limbs is often high. This paper faces this issue, by proposing and experimentally assessing a set of algorithms which are capable of proportionally and simultaneously control as many actuators as there are independent electromyographic signals available. Two sets of solutions are considered. The first uses as input electromyographic signals only, while the second adds postural measurements to the sources of information. At first, all the proposed algorithms are experimentally tested in terms of precision, efficiency, and usability on twelve able-bodied subjects, in a virtual environment. A state-of-the-art controller using twice the amount of electromyographic signals as input is adopted as benchmark. We then performed qualitative tests, where the maps are used to control a prototype of upper limb prosthesis. The device is composed of a robotic hand and a wrist implementing active prono-supination movement. Eight able-bodied subjects participated to this second round of testings. Finally, the proposed strategies were tested in exploratory experiments involving two subjects with limb loss. Results coming from the evaluations in virtual and realistic settings show encouraging results and suggest the effectiveness of the proposed approach.

## 1. Introduction

Since its first appearance in the '40s (Leon Gillis, 1948), myoelectric control has established itself as an effective mean of controlling artificial limbs. Nowadays, its range of application extends to all those fields of robotics in which humans have to control robots, as teleoperation (Vogel et al., 2011; Meeker and Ciocarlie, 2018), assistive robotics (Song et al., 2008), supernumerary limbs (Hussain et al., 2016; Leigh and Maes, 2016; Ciullo et al., 2018), and of course prosthetics (Segil et al., 2014; Godfrey et al., 2018; i limb, 2018; Michelangelo, 2018; Taska, 2018; Vincent, 2018).

To control the large number of degrees of freedom typically present in artificial limbs and hands, a considerable amount of muscular signals are needed. This represents a major challenge in developing usable EMG-based control interfaces. This issue becomes even more compelling when the user is impaired by an amputation, by a stroke, or by other pathologies. In these situations, indeed, the number of muscles from which it is possible to extract clean and independent signals in general is very small. As an example, we report in Figure 2 the results of an experimental analysis we performed on data acquired from an impaired subject (see section 6). The EMG data acquired from the stump is embedded (90% of the total variance) in a space of dimension two, so only two independent features can be extracted from this source of information. For reference, consider that the sound arm produces activation patterns with higher dimensionality (usually around four). Thus, toward the prosthetic application, it is paramount to devise control algorithms making the most out of this low number of EMG features.

Most commercial solutions used today in prosthetics deal with this problem by managing the degrees of actuations (DoA) of the prosthesis through switching techniques, as co-contractions, sequential activation, mechanical selectors, software applications for smart phones, just to cite a few (Roche et al., 2014). While effective in enabling the exploitation of a large number of actuators, these solutions are considered uncomfortable by many users, often leading to abandonment of the device (Biddiss and Chau, 2007). Classification algorithms aim at providing a more natural control interface. Here, features extracted from the residual muscular activation are fed into a classifier, which is trained to recognize hand postures or grasp patterns. Once the system recognizes a memorized category, the device is commanded to move as to reproduce the associated posture. Notable examples are Huang et al. (2005), Hargrove et al. (2007), Jiang et al. (2012a), and Batzianoulis et al. (2018). The use of postural information is considered in Krasoulis et al. (2017) for improving classification performance, and in Bennett and Goldfarb (2017) for controlling an active wrist while the hand posture is specified by the classifier. These techniques represent a clear improvement with respect to the switching policies. Nevertheless, they are still limited in terms of naturalness of use, since they allow for a single matching per activation, often leading to unnatural transitions.

The best performances in terms of usability are provided by proportional controllers. Here control features extracted from electromyographic signals are directly mapped into the motor reference positions, with the goal of achieving an intuitive and direct mapping of user's intentions in motions of the artificial limb (Choi and Kim, 2011; Jiang et al., 2012b; Belyea et al., 2018; Schmalfuss et al., 2018). However, these performance come at the cost of requiring to extract more than one EMG signal for each degree of actuation. This, in the practice, strongly restricts the usability of proportional techniques.

This work tackles this issue, with specific focus on the surface electromyographic sensors case (Navarro et al., 2005; Cipriani et al., 2011). We propose six simple, yet effective, maps that enable controlling as many DoA as the number of independent signals extracted from the muscles. Three out of six use only electromyographic signals, while the other three integrate muscular information with postural measurements. This goal is achieved by using non-linear filters to process the signals.

The proposed algorithms are first extensively tested into a simulated environment and compared with a standard approach using twice the electromyographic measurements, as an upper bound benchmark. During these experiments, twelve able-bodied subjects were asked to control a pointer on a screen by activating muscles of their upper limb. We introduce a set of objective metrics inspired from (Williams and Kirsch, 2008; Scheme et al., 2014), and a Likert-like questionnaire (Likert, 1932) to be filled by the subjects. In this way it was possible to assess maps precision, accuracy, and usability.

**Figure 1:**
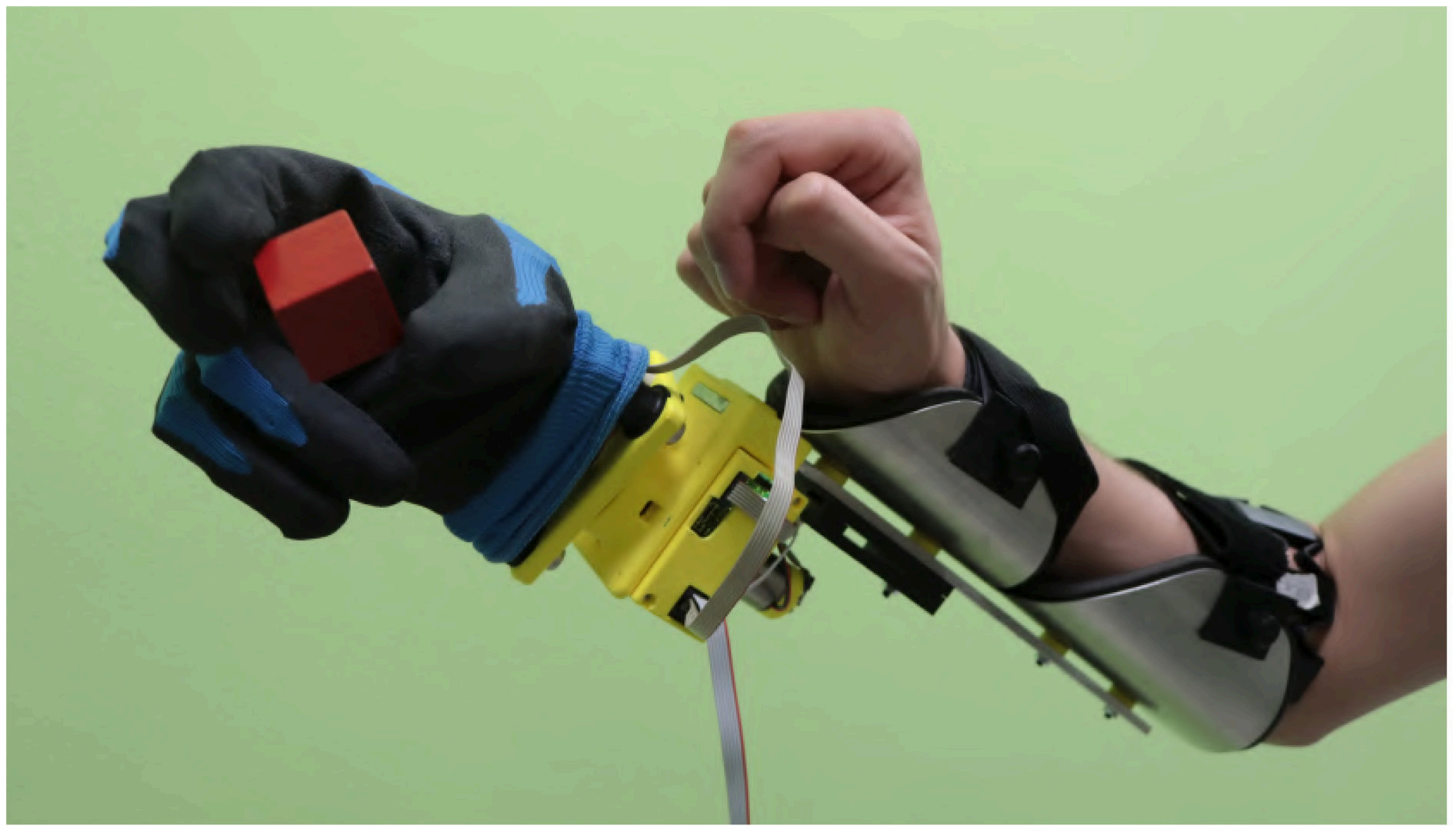
The considered multi-modal extra-limb, grasping a wooden cube. An operator controls the device through independent contraction of extensor and flexor carpi muscles, and through forearm posture.

With the aim of qualitatively evaluating the algorithms in operating a physical device, we introduce the prototype in Figure 1. We derive it from Pisa/IIT SoftHand 2 (Della Santina et al., 2018), an underactuated hand with 19 DoF and 2 DoA implementing the most common movements of the human hand (Della Santina et al., 2017a) as free motions. The EMG control of this robotic hand was preliminarily tested in (Rossi et al., 2017), with promising results. Motivated by several studies discussing the importance of wrist motions in task execution (Bajaj et al., 2015; Montagnani et al., 2015b; Merad et al., 2018), we complete the device with the introduction of an active wrist prototype, implementing prono-supination motions. The two algorithms that performed best in virtual environment were tested together with the benchmark by eight able-bodied subjects performing three standardized tasks; (i) moving small wooden blocks from a box to another, (ii) building a pyramid with the same blocks, (iii) turning cards. Evaluations scales are introduced to quantitatively assess both performance and usability. The system is also operated by an expert user in the execution of further daily life activities. The combination of intelligence embodied in the soft device and the control maps proposed here, enables the subject to naturally perform complex actions.

**Figure 2:**
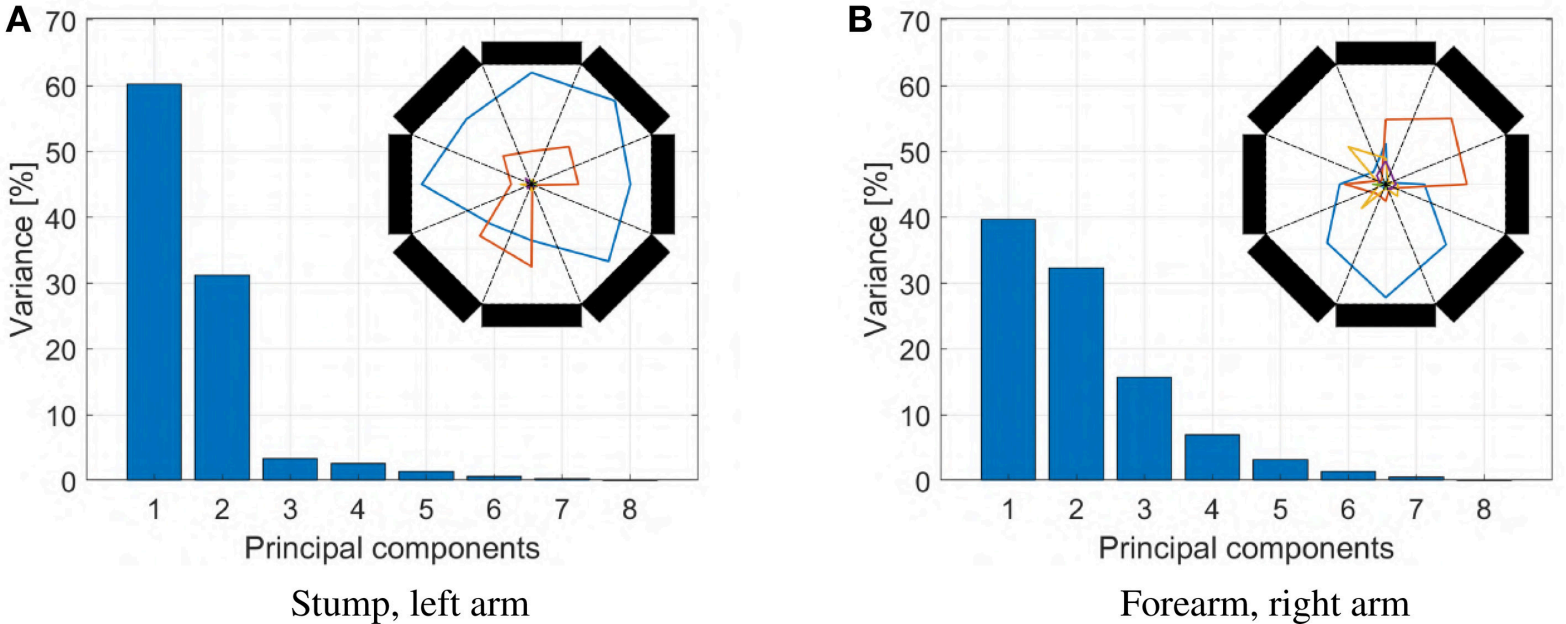
Results of a dimensionality analysis performed on data acquired from an impaired subject (Subject 1 in section 6). The subject has congenital malformation at the trans-radial level in the left hand. Data were acquired by placing a MYO Armband (see section 4) on the stump, and another on the sound arm, approximatively at the same distance from the elbow. The subject is then asked to move the right hand, and imagining to move the missing hand, to four reference postures (prono-supination of the wrist, flexo-extension of the wrist, adduction-abduction of the wrist, opening and closing of the hand). The subject had past experience with this kind of experiments, so a short amount a pre-training was sufficient. The EMG data so acquired are analyzed using principal component analysis technique. The dimensionality of electromyographic signals coming from the stump are shown in panel **(A)**, and the ones coming from the sound arm are shown in panel **(B)**. The EMG signals coming from the stump are clearly embedded in a space of dimension two. On the contrary, the ones coming from the sound arm are more evenly distributed. We report in the top right of each figure the radar chart showing the absolute values of the activations corresponding to the principal components extracted. The amplitudes are proportional to the explained variances.

Finally, exploratory studies were carried out, involving two subjects with limb loss. The results are promising, further proving the experimental effectiveness of the proposed strategies.

The work is organized as follows. Section 2 provides an overview on the problem of extracting interested features from a residual upper-limb. In section 3 we introduce the proposed approach by describing the two sets of control maps. In section 4 we analyze the proposed controllers in the virtual environment, and in section 5 with the prosthetic prototype. Section 6 reports the results of experiments with impaired subjects.

## 2. Problem Statement

### 2.1. Background: Standard Control Pipeline

**Figure 3:**
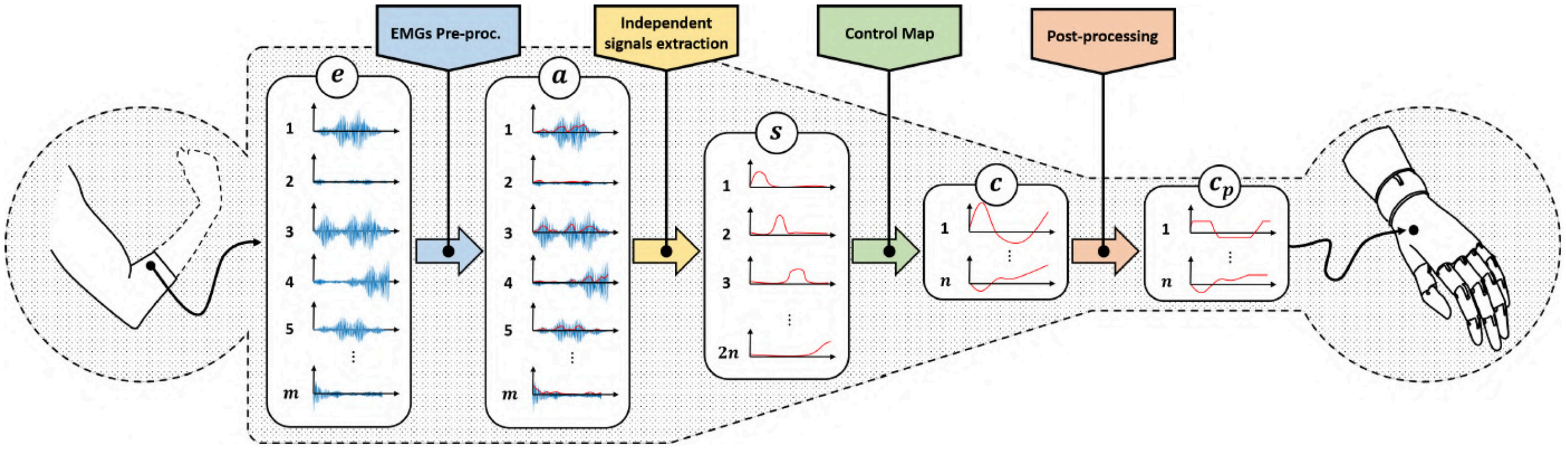
Classical control architecture of a myoelectric device using proportional approach. We refer with *e* ∈ ℝ^*m*^ to the acquired EMG signals, $a\in {\mathbb{R}}_{+}^{m}$ represents the residual muscular activations, $s\in {\mathbb{R}}_{+}^{2n}$ are the independent signals obtained by factorization, *c* ∈ ℝ^*n*^ is the set of control signals obtained by the application of a proportional control map and $c_{p}\in {\mathbb{R}}^{n}$ are the control signals adapted to the specific device characteristics.

Figure 3 depicts a standard architecture for proportional myoelectric control. The first layer of the architecture rectifies and filters the EMG signals *e* ∈ ℝ^*m*^ read by sensors (De Luca et al., 2010). The output $a\in {\mathbb{R}}_{+}^{m}$ is a measure of the levels of muscular activation. Note that each element of *a* is strictly positive, as expected when considering the nature of the measured phenomenon.

These signals are in general redundant, in the sense that a reduced amount of muscles, or muscle synergies (d'Avella et al., 2003), is often measured by a large amount of sensors. So, the second layer uses factorization techniques (Choi and Kim, 2011; Jiang et al., 2014) to extract the ideally maximum amount of independent features $s\in {\mathbb{R}}_{+}^{2n}$, where 2*n* ≤ *m*. For the sake of simplicity, we assume the number of independent features to be even.

The following layer of the pipeline maps the continuous and independent features *s* into commands to be sent to the artificial limb *c*. Note that the main challenge preventing a simple solution is that *s* lives into ${\mathbb{R}}_{+}^{2n}$, i.e., it is strictly positive. *c* instead assumes both positive and negative values. A very simple way for achieving this goal is (Scott and Parker, 1988)

(1) $$\begin{array}{r} c_{i}=s_{2i-1}-s_{2i}, \end{array}$$

where *c*_*i*_ and *s*_*i*_ are the j-th control signal and feature, respectively. (*s*_2*i*−1_, *s*_2*i*_) are often selected as the independent signals extracted by a couple of antagonistic muscles. This very simple linear control map already enables the mapping of two positive signals in a signal spanning all ℝ, with the useful feature of automatically getting rid of co-activations^1^ typically generated by inexpert users (Scheme and Englehart, 2011). The control strategy 1 is very natural for an user, since it mimics what happens with a standard human joint i.e., that a pair of muscles actuate a single joint.

Equation 1 can be generalized as

(2) $$\begin{array}{r} c=A^{\left(i\right)}\left(s\right)\;:\;{\mathbb{R}}_{+}^{2n}\to {\mathbb{R}}^{n}, \end{array}$$

where we stress that this class of methods requires independent signals in twice the number of the DoA to be controlled.

Finally, some post-processing (e.g., saturations, normalizations) concludes the pipeline. The output signals *c*_p_ are directly commanded to the motors of the artificial limb.

### 2.2. Problem: Mapping From Many to Few

As shown in Figure 3, two reductions of the size of the signals occur through the pipeline. The first one is operated by the independent signal extraction. We consider here ideal performance for this layer. A large part of the current research in EMG control is indeed focused on improving it. We refer the interested reader to (Farina et al., 2014) for a in depth analysis of the state of the art and future challenges in this topic. We thus hypothesize the dimensionality of *s* to be the maximum possible, and that the reduction in size is only to remove redundancy from the signals.

**Figure 4:**
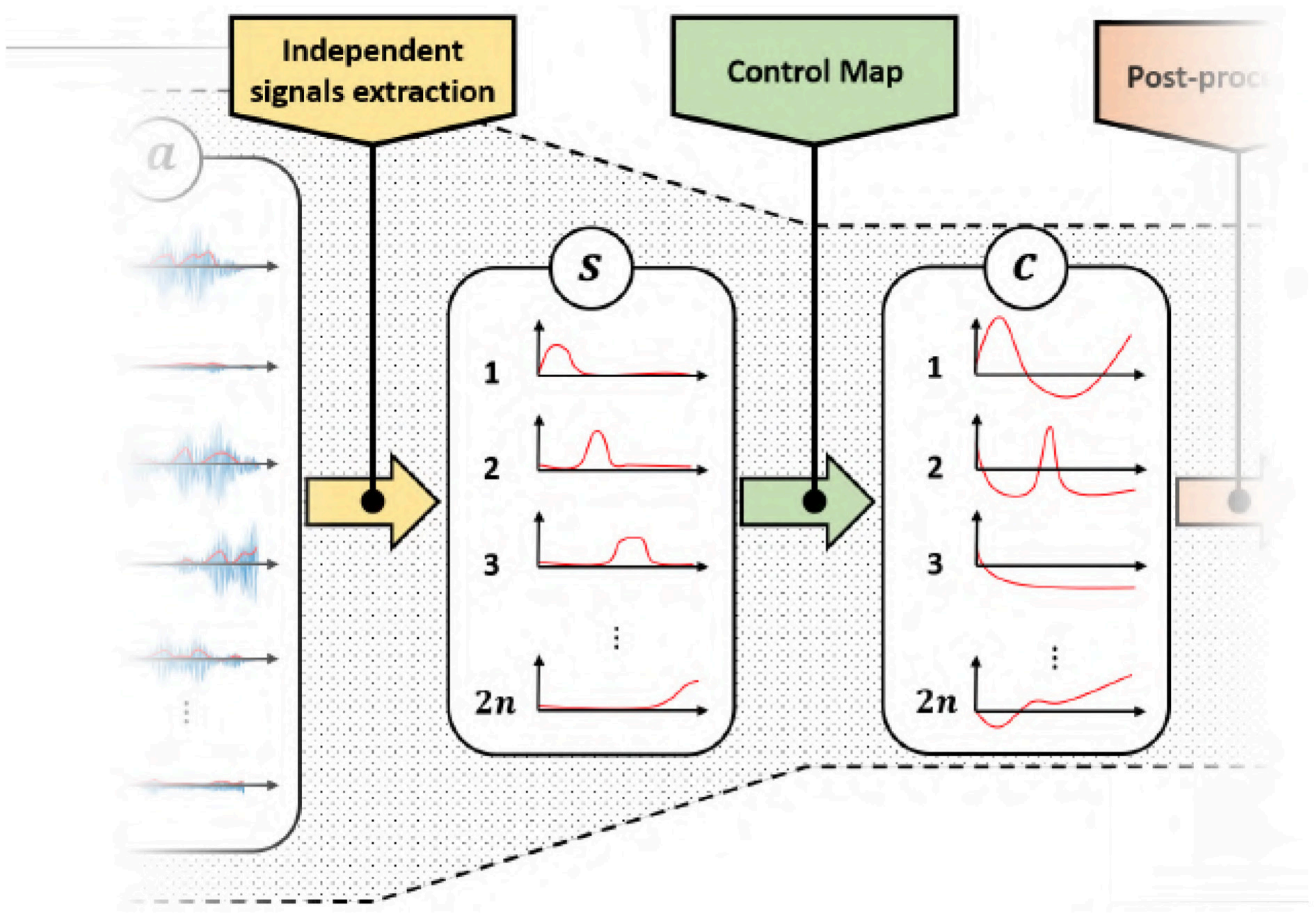
Modified control architecture using the proposed control maps with only EMG signals as input. Using the proposed approach we have twice the number of control signals *c* of the classical approach (Figure 3). $a\in {\mathbb{R}}_{+}^{m}$ represents the residual muscular activations, $s\in {\mathbb{R}}_{+}^{2n}$ are the independent signals obtained by factorization, *c* ∈ ℝ^*n*^ is the set of control signals obtained by the application of a proportional control map.

**Figure 5:**
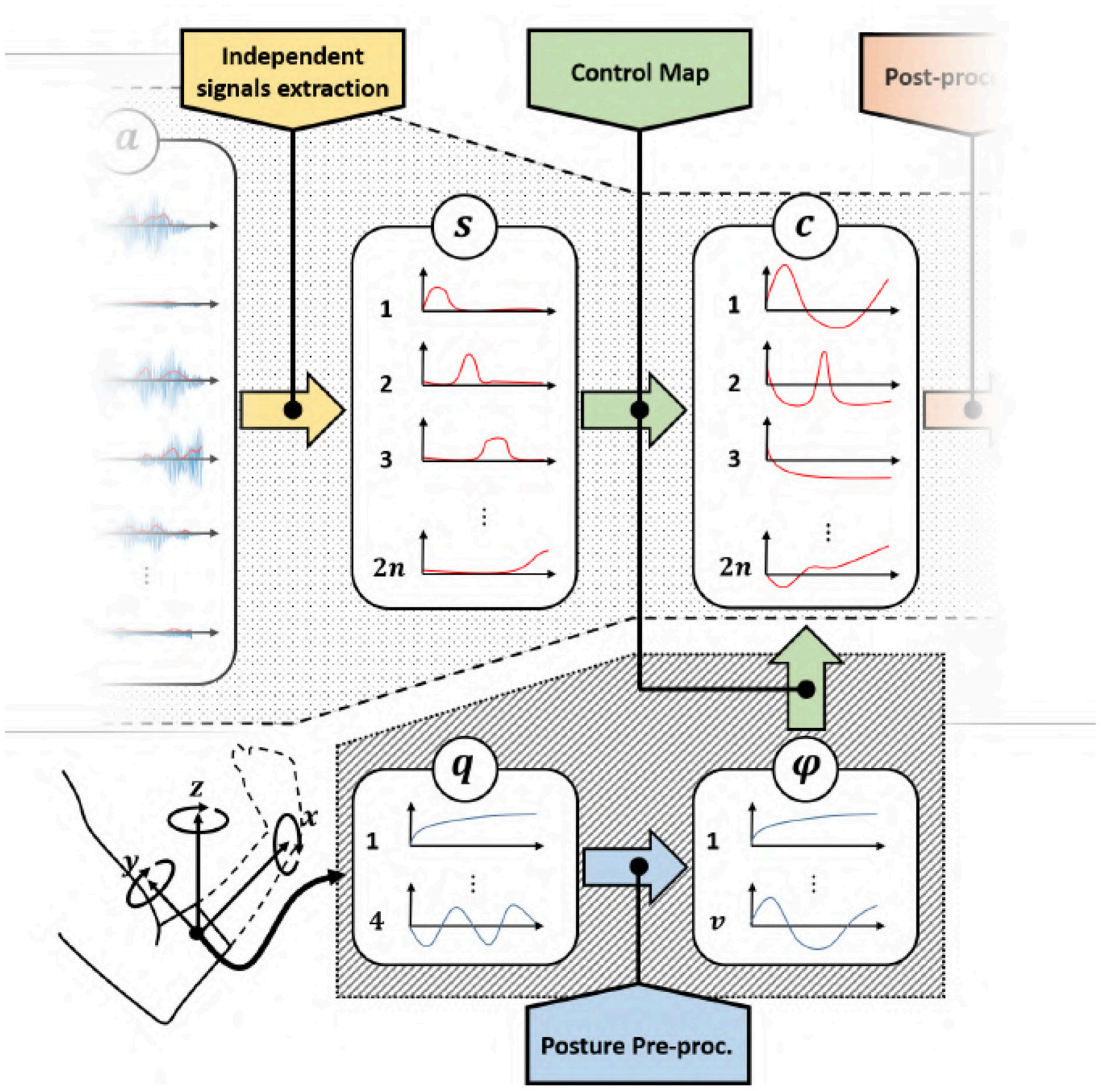
Modified control architecture using the proposed control maps with EMG signals and postural information as input. Also in this case we achieve two times the number of control signals *c* than the classical approach (Figure 3), with a same number of EMG signals. $a\in {\mathbb{R}}_{+}^{m}$ represents the residual muscular activations, $s\in {\mathbb{R}}_{+}^{2n}$ are the independent signals obtained by factorization, *c* ∈ ℝ^*n*^ is the set of control signals obtained by the application of a proportional control map, *q* is the orientation of the forearm in quaternion representation and ϕ are the extracted angular features.

The rest of the paper will focus on the second bottleneck. We will design and validate novel approaches to map features *s* into commands *c*. Two sets of control map will be devised, each one enabling the control of 2*n* DoAs when only 2*n* independent EMG signals are available. The first set of maps is called EMGs-only (EMGs-MAPs) and uses only EMG signals as inputs (Figure 4). We then extend the analysis by considering the addition of external signals such as, for example, posture information. This set is called augmented map (AUG-MAPSs) (Figure 5).

Before moving to maps definition, it is worth mentioning that mathematically it is always possible to solve the problem by defining a function

(3) $$\begin{array}{r} A^{\left(ii\right)}\left(s\right)\;:\;{\mathbb{R}}_{+}^{2n}\to {\mathbb{R}}^{2n}, \end{array}$$

with *A*^(*ii*)^(*s*) surjective. Yet, this can be done only using discontinuities, asymptotes or other mathematical constructs that make the system difficult to control. Simple examples are log(*x*) and $\frac{1-x^{2}}{x}$. We will thus not consider this class of strategies further in this paper. A simpler solution could be found in

(4) $$\begin{array}{r} A^{\left(ii\right)}\left(s\right)=s-\bar{s}, \end{array}$$

where $\bar{s}\in {\mathbb{R}}_{+}^{2n}$ is a positive constant. This map would of course not be surjective. Nonetheless, it is in principle sufficient to include in the co-domain all the values of *s* that map into the finite range of the actuators. This can be achieved by choosing $\bar{s}$ large enough. However, this solution has two main drawbacks. The first is shared to all the solutions in the form 3, and it is that purely algebraic maps tend to tire the user after very little time. Indeed, to maintain a constant configuration of the artificial limb, they would have to generate a constant contraction of the muscles. The second drawback is specific of 4. We observed that having the null muscle activation (i.e., *s* = 0) mapped to a non-neutral configuration (i.e., $c=\bar{s}$) was very unnatural for the user. This sensibly reduced the acceptance of the artificial limb, and strongly increased the effort required for piloting it. We therefore add as further constraint that our maps must fulfill, that a null muscle activation should not move the artificial limb from its neutral configuration (*c* = 0). Thus, the proposed map must have the origin as fixed point.

### 2.3. Proposed Solution: Using Non-linear Filtering to Design Control Maps

#### EMGs-MAPs

These maps aim at obtaining and higher level of usability, by extracting more information from the available signals. Looking at the literature, the control map 1 is often modified to increase its robustness and usability into

(5) $$\begin{array}{r} c_{j}=\overset{t}{\underset{0}{\int }}\left(s_{2j-1}-s_{2j}\right)\text{d}t. \end{array}$$

This simple, yet effective strategy still maps ${\mathbb{R}}_{+}^{2n}$ into ℝ^*n*^. However, it suggests us the possibility of exploiting the temporal evolution of the signal *s* to enrich the information to be mapped into *c*. We thus generalize 5 as the generic continuous non-linear filter

(6) $$\{{}_{c=A^{\left(iii\right)}(s,x)}^{\dot{x}=f(s,x)}\;,$$

where $A^{\left(iii\right)}:{\mathbb{R}}_{+}^{2n}\times {\mathbb{R}}^{w}\to {\mathbb{R}}^{2n}$, $f:{\mathbb{R}}_{+}^{2n}\times {\mathbb{R}}^{w}\to {\mathbb{R}}^{w}$, and *x* ∈ ℝ^*w*^ is the state of the filter encoding the extracted temporal information.

#### AUG-MAPs

We consider here external signals in addition to EMGs (Figure 5). In this case 6 can be easily generalized by adding a new set of inputs

(7) $$\{{}_{c=A^{\left(iv\right)}(s,p,x)}^{\dot{x}=f(s,p,x)}\;,$$

where $A^{\left(iv\right)}:{\mathbb{R}}_{+}^{2n}\times {\mathbb{R}}^{k}\times {\mathbb{R}}^{w}\to {\mathbb{R}}^{2n}$, $f:{\mathbb{R}}_{+}^{2n}\times {\mathbb{R}}^{w}\times {\mathbb{R}}^{k}\to {\mathbb{R}}^{w}$, 2*n* is the number of independent EMG signals, *k* is the number of external inputs, and *w* is the number of temporal features.

A useful source of information usable as external input is the position and orientation of the residual forearm. Figure 5 shows a control scheme with posture as additional input, where *q* is the orientation of the forearm in quaternion representation, and ϕ are the extracted angular features.

## 3. Maps Definition

Among all the possible non-linear filters satisfying the definitions 6 and 7, we propose here six maps which we expect to present a natural and effective interface to the user. We will extensively test these aspects in the next sections. In analogy to 1 and 5—and with the aim of simplifying the user interface—we propose maps elaborating on the input signals in a pairwise manner. For the sake of simplicity of notation we call the two input signals *s*_1_, *s*_2_, and the two outputs *c*_1_, *c*_2_. This choice also helps us in graphically interpreting the map behavior, since this sub-mapping can be seen as operating a transformation from the first quadrant (plus time), to the whole ℝ^2^ plane. A generic ${\mathbb{R}}_{+}^{2n}$ to ℝ^2*n*^ map can be realized by replicating *n* times the algorithms proposed here. The assessment of this case will be addressed in future work.

### 3.1. EMGs-Only Maps (EMGs-MAPs)

#### Map 1:

Leveraging the geometric interpretation of *c* as a point in a two dimensional plane, we consider as first attempt to designing a square map, the change of coordinates from polar to Cartesian. A direct implementation would consist in using one feature as magnitude and the other as angle, i.e.,

(8) $$\begin{array}{r} \left[\begin{array}{c} c_{1}\\ c_{2} \end{array}\right]=s_{1}\left[\begin{array}{c} \sin\left(s_{2}\right)\\ \cos\left(s_{2}\right) \end{array}\right]. \end{array}$$

However, such a choice appeared in exploratory evaluations particularly unnatural to the users. We individuated three main issues causing this, in addition to the one discussed in general for 3; (i) when *s*_1_ is large, very small oscillations in *s*_2_ produce large variations of (*c*_1_, *c*_2_); (ii) moving from (*c*_1_, *c*_2_) both positive to *c*_1_ positive and *c*_2_ negative requires to pass first through *c*_1_ negative and *c*_2_ positive, and then through (*c*_1_, *c*_2_) both negative; (iii) in the common case of (*s*_1_, *s*_2_) associated to two antagonistic muscles, generating the two activations independently is very demanding for the user. Overall these three effects produce a very shaky behavior in terms of device configuration, with unnatural and unexpected motions.

To overcome the limitation (iii) we consider the magnitude of the position defined as the input semi-sum

(9) $$\begin{array}{r} p=\frac{s_{1}+s_{2}}{2}. \end{array}$$

The phase rate instead is obtained by the semi-difference between input signals

(10) $$\begin{array}{r} \beta =\frac{s_{1}-s_{2}}{2}. \end{array}$$

We indeed observed that it was rather natural for an user to associate co-contraction to the module of (*c*_1_, *c*_2_), and the semi-difference to the angle. This also solves problem (ii) since β ∈ ℝ. Nonetheless, this choice risks to exacerbate (i). Following the example of 5, we apply an integration to β to generate a more stable and firm behavior.

So, the resulting non-linear filter is

(11) $$\{{}_{\left[{}_{c_{2}}^{c_{1}}\right]\;=\;\frac{s_{1}+s_{2}}{2}\;\left[{}_{\cos(x_{1})}^{\sin(x_{1})}\right]\;.}^{\;{\dot{x}}_{1}\;=\;\frac{s_{1}-s_{2}}{2}}$$

**Figure 6:**
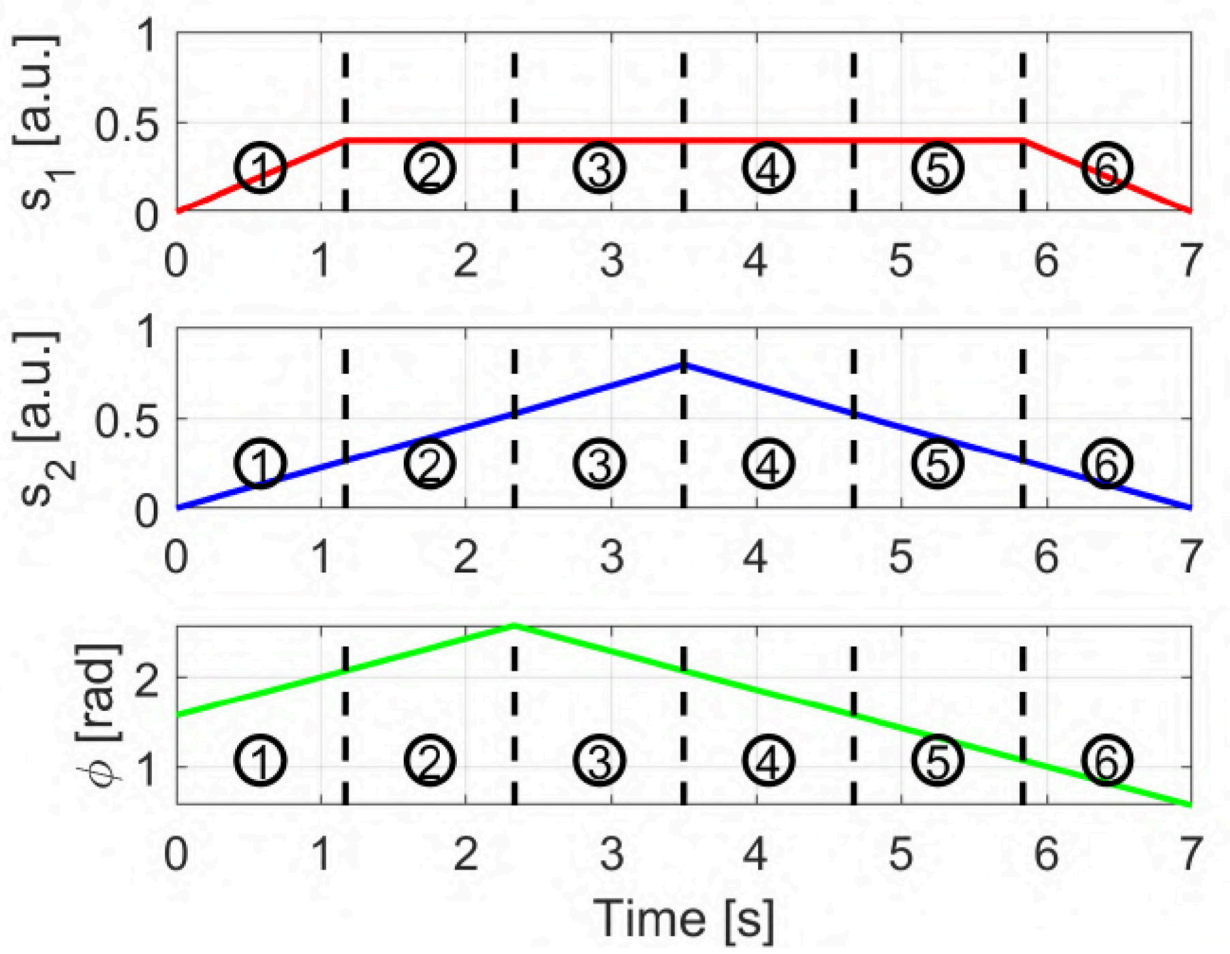
Input signals used to obtain the sequences displayed in Figures 7, 8. *s*_1_ and *s*_2_ are the two independent signals, with *s*_*i*_ ∈ [0 1]. ϕ represents the torsion angle used in AUG-MAPs, where ϕ ∈ [−π, π].

**Figure 7:**
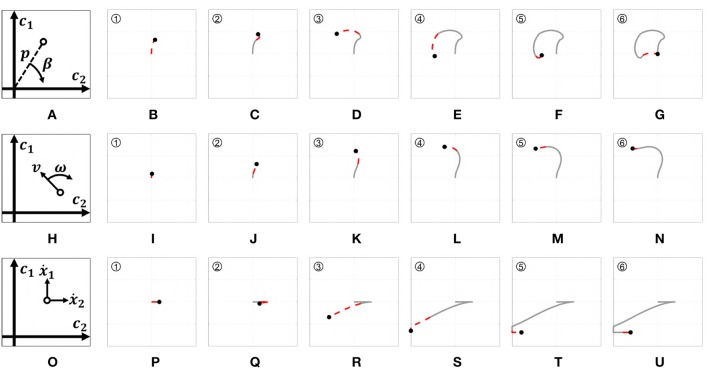
Behavior of the three EMGs-MAPs in response to a same input pattern (see Figure 6). Panels **(A,H,O)** sketch the functioning of the maps 1–3, respectively. Panels **(B–G)** show the output corresponding to Map 1, **(I–N)** to Map 2, **(P–U)** to Map 3. The portion of the lines which is red and dashed highlights the path that it is executed during that time portion (indicated by the number on the top left).

**Figure 7A** provides a graphic representation of this control strategy. **Figures 7B–G** show the output *c* when the input *s* shown in Figure 6 is used.

#### Map 2:

By adding to 11 a second level of integration we obtain the following non-linear system

(12) $$\begin{array}{ll} \left\{ \begin{array}{l} \left[\begin{array}{c} {\dot{x}}_{1}\;\\ {\dot{x}}_{2}\;\\ {\dot{x}}_{3} \end{array}]\;=\;[\begin{array}{c} \omega \\ v\sin(x_{1})\\ v\cos(x_{1}) \end{array}\right]\\ \left[\begin{array}{c} c_{1}\\ c_{2} \end{array}]\;=\;[\begin{array}{c} x_{2}\\ x_{3} \end{array}\right] \end{array}\right., & \;\left(12\right) \end{array}$$

where $v=\frac{s_{1}+s_{2}}{2}$ and $\omega =\frac{s_{1}-s_{2}}{2}$. In this way we aim at achieving a more stable behavior during task execution.

This non-linear system got a lot interest in the early years of geometric non-linear control (Brockett, 2014). It models indeed an unicycle (Aicardi et al., 1995) with ω and *v* being the angular and linear velocities, respectively. Several works proved that by opportunely using ω and *v* all the points in ℝ^2^ can be reached, even in presence of constraints on ω, and with strictly positive *v* (Dubins, 1957; Consolini et al., 2009). This assures that the map is well-posed.

Figure 7H provides a graphic representation of this control strategy. Figures 7I–N show the output *c* when the input *s* shown in Figure 6 is used.

#### Map 3:

Several studies have shown that humans tend to execute different kinds of movements on different time scales (Vainio et al., 2008). An example is the precision grasp, which is typically performed in long time scales, and the power grasp, which is instead typically performed rapidly. Moving from this consideration, (Piazza et al., 2016) proposed exploiting the temporal information encoded in the control signal to choose the movement to perform with a prosthetic hand. That idea was there mechanically implemented through the use of damping elements. In that way, the type of grasp changed depending on the velocity of closure.

We propose here to exploit the same idea in mapping control signal to a generic artificial limb. To implement this behavior we set the velocity on each output axis proportional to the activation signals. The sign of each output channel depends on which independent signal is higher. Then, we use thresholds to discriminate high activations from small ones. More specifically, movement on *c*_2_ will occur for small activations only, whereas on both *c*_1_ and *c*_2_ will with high activations.

(13) $${\dot{x}}_{1}=\{\begin{array}{cc} k_{i}(s_{1}-a_{i}) & \text{if }(s_{1}>s_{2})\wedge (s_{1}>a_{i})\\ -k_{i}(s_{2}-a_{i}) & \text{if }(s_{1}<s_{2})\wedge (s_{2}>a_{i})\\ 0 & \text{otherwise} \end{array}$$

and

(14) $$\begin{array}{r} c_{i}=x_{i}, \end{array}$$

with *i* ∈ {1, 2}. *a*_1_ and *a*_2_ are two different activation thresholds such that *a*_2_ < *a*_1_. Figure 7O provides a graphic representation of this control strategy. Figures 7P–U show the output *c* when the input *s* shown in Figure 6 is used.

### 3.2. Augmented Maps (AUG-MAPs)

AUG-MAPs use external inputs to overcome the lack of information provided by the sEMG. Ideally, we could use any type of signal as external input. One interesting source is the posture of the forearm, which holds information about the subject's intentions of movement (Montagnani et al., 2015a). The following maps use as external input the torsion angle ϕ ∈ ℝ of the shoulder extracted from the forearm posture. This signal is used in addition to the same two independent signals used in the first three maps.

#### Map 4:

**Figure 8:**
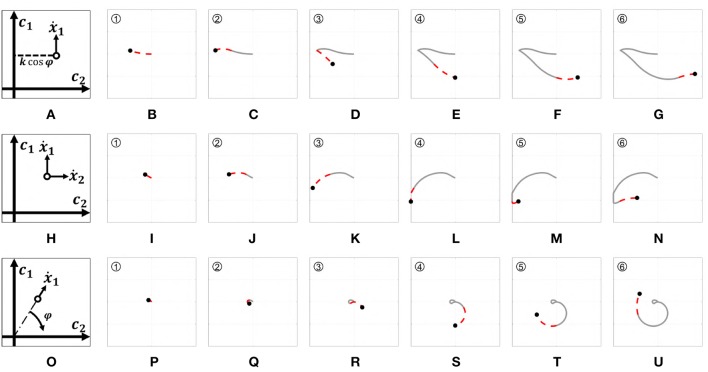
Behavior of the three EMGs-MAPs in response to a same input pattern (see Figure 6). Panels **(A,H,O)** sketch the functioning of the maps 4–6, respectively. Panels **(B-G)** show the output corresponding to Map 4, **(I-N)** to Map 5, **(P-U)** to Map 6. The portion of the lines which is red and dashed highlights the path that it is executed during that time portion (indicated by the number on the top left).

A simple way to use an external signal to directly map it onto one output channel. EMG signals are then used to activate the other channel. More in detail, this map regulates the rate of activation of the first channel as the semi-difference between the EMG signals. The activation of the second channel instead is proportional to the cosine of the torsion angle. So, we define the first augmented map as

(15) $$\begin{array}{ll} \left\{ \begin{array}{l} {\dot{x}}_{1}=\frac{s_{1}-s_{2}}{2}\\ \left[\begin{array}{l} c_{1}\\ c_{2} \end{array}]=[\begin{array}{c} x_{1}\\ k\cos\phi \end{array}\right], \end{array}\right. & \;\left(15\right) \end{array}$$

where *k* is a normalization constant. Figure 8A presents a graphic representation of how this control works. Figures 8B–G show the outputs behavior obtained applying to this map the input signals shown in Figure 6.

#### Map 5:

Adding a level of integration to the previous map we obtain another unicycle-like system. The rate of activation on the first channel is activated by the semi-difference between the EMG signals. The torsion angle, instead, regulates the rate of activation on the second channel. The resulting map is

(16) $$\{{}_{[\begin{array}{c} c_{1}\\ c_{2} \end{array}]\;=\;[\begin{array}{c} x_{1}\\ x_{2} \end{array}]\;.}^{\left[\begin{array}{c} \;{\dot{x}}_{1}\\ \;{\dot{x}}_{2} \end{array}]\;=\;[\begin{array}{c} \frac{s_{1}-s_{2}}{2}\\ k\cos\phi \end{array}\right]}$$

Figure 8H presents a graphic representation of how this control works. Figures 8I–N show the outputs behavior obtained applying to this map the input signals shown in Figure 6.

#### Map 6:

Considering the input as polar coordinates it is possible to create a direct connection between the torsion angle ϕ and the phase angle of the output coordinates. Concretely, the variation of magnitude is defined as the semi-difference between the EMG signals, while the phase is proportional to the torsion angle. The resulting augmented polar map is

(17) $$\begin{array}{ll} \left\{ \begin{array}{l} \;{\dot{x}}_{1}\;=\;\frac{s_{1}-s_{2}}{2}\\ {}[\begin{array}{l} c_{1}\\ c_{2} \end{array}]\;=\;kx_{1}\;[\begin{array}{c} \sin(\phi )\\ \cos(\phi ) \end{array}]\;. \end{array}\right. & \;\left(17\right) \end{array}$$

Figure 8O presents a graphic representation of how this control works. Figures 8P–U show the outputs behavior obtained applying to this map the input signals shown in Figure 6.

## 4. Usability Assessment in Virtual Environment

To validate the quality of control algorithms independently from the performance of the controlled device, it is common in the literature to develop and use a virtual testing environment (Williams and Kirsch, 2008; Scheme et al., 2014). We follow here the same approach for validating and comparing the proposed maps in terms of precision, accuracy and users' appreciation.

### 4.1. Benchmark

To have a fair comparison of the results, in addition to the proposed maps we considered a benchmark.

#### Map 7:

This map uses four independent signals combined as described in Equation 5. Each pair of antagonistic signals is used to activate a different DoA. So, we define the benchmark (BM) map as

(18) $$\{{\;}_{[\begin{array}{c} c_{1}\\ c_{2} \end{array}]\;=\;[\begin{array}{c} x_{1}\\ x_{2} \end{array}]\;,}^{\left[\begin{array}{c} \;{\dot{x}}_{1}\\ \;{\dot{x}}_{2} \end{array}]\;=\;[\begin{array}{c} s_{1}-s_{2}\\ s_{3}-s_{4} \end{array}\right]}$$

where *s*_1_, *s*_2_, *s*_3_, *s*_4_ are roughly connected to flexion, adduction, extension, abduction wrist movements. It is worth noticing that the benchmark is in a favorable position w.r.t. the proposed maps. Indeed, this algorithm uses twice the number of EMG independent signals (i.e., $s\in {\mathbb{R}}_{+}^{4}$) w.r.t. our maps, to produce a same number of outputs (*c* ∈ ℝ^2^). Note also that the count is in favor of the benchmark, even by adding to it ϕ ∈ ℝ—which we should not since this signal is not affected by the availability problem discussed in the Introduction section. So, the benchmark is to be intended as an upper bound to the performance achievable by the maps, rather than a lower bound to beat.

### 4.2. Experimental Setup

**Figure 9:**
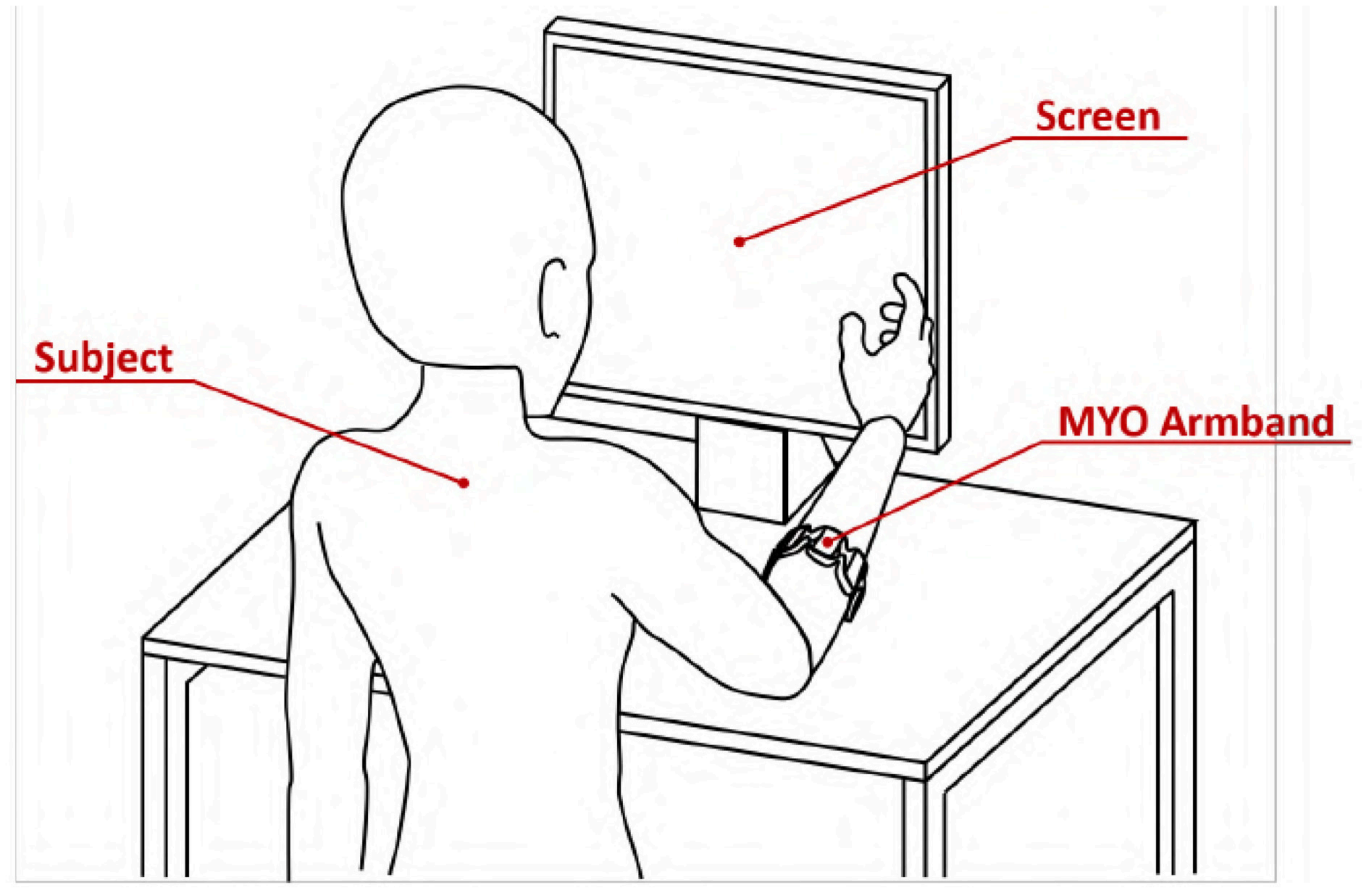
Virtual environment validation experimental apparatus. The subject was asked to wear the MYO Armband on the right forearm near the elbow. The subject was instructed to seat in front of the screen with the right arm in a comfortable position.

The experimental setup, portrayed in Figure 9, is composed of

- **Virtual environment**: the output signal (*c*_1_, *c*_2_) is represented as a point on a two dimensional space, visualized on a dedicated screen. Targets of different sizes can be added to the scene. This environment was implemented via Simulink and MatLab on a personal computer.
- **MYO Armband**: for the acquisition of the input signals we used a MYO Armband ^2^. It consists of a bracelet with 8 EMG sensors evenly spaced. Each sensor provides a 200Hz EMG signal (*e*_1_, …, *e*_8_) with a resolution of 8 bits. This device is also equipped with an IMU placed on block 4. The IMU module provides a 50Hz channel containing the quaternion of the device orientation. Each element of the quaternion is represented with two bytes. The MYO Armband needs no cable connection. The device uses a BLE Bluetooth module to communicate with other devices. Please refer to (Pizzolato et al., 2017) for an exhaustive discussion about the use of this device in prosthetics.

The extraction of *s* from (*e*_1_, …, *e*_8_) is performed in accord with the standard pipeline of section 2.1, implemented through the algorithms described in Appendix. It is important to remark here that these eight signals are not independent, as a lot of redundancy is present. More specifically—as proven by Figure 2—the amount of independent features that can be extracted from *e* is about four for an able-bodied subject, and around two for a subject with limb loss.

### 4.3. Participants

Twelve able-bodied subjects took part in the experiment, six men and six women aged from 23 to 31. All subjects were tested on their dominant hand (right hand; self-reported hand dominance). All participants were naive to the experimental purpose of the study and had no history of neuromuscular disorders. Before data collection, subjects signed an informed consent to participate in the experiment. The experimental protocols were approved by the Institutional Review Board of University of Pisa, in accordance to the Declaration of Helsinki.

### 4.4. Experiment

Subjects were asked to wear the MYO Armband on the right forearm near the elbow. They were instructed to seat in front of the screen with the right arm in a comfortable position, as shown by Figure 9.

During the experiment subjects were requested to navigate the virtual plane by applying the right combination of muscular activations. Clearly, the combination of inputs moving the pointer in a specific direction depends on the map used.

**Figure 10:**
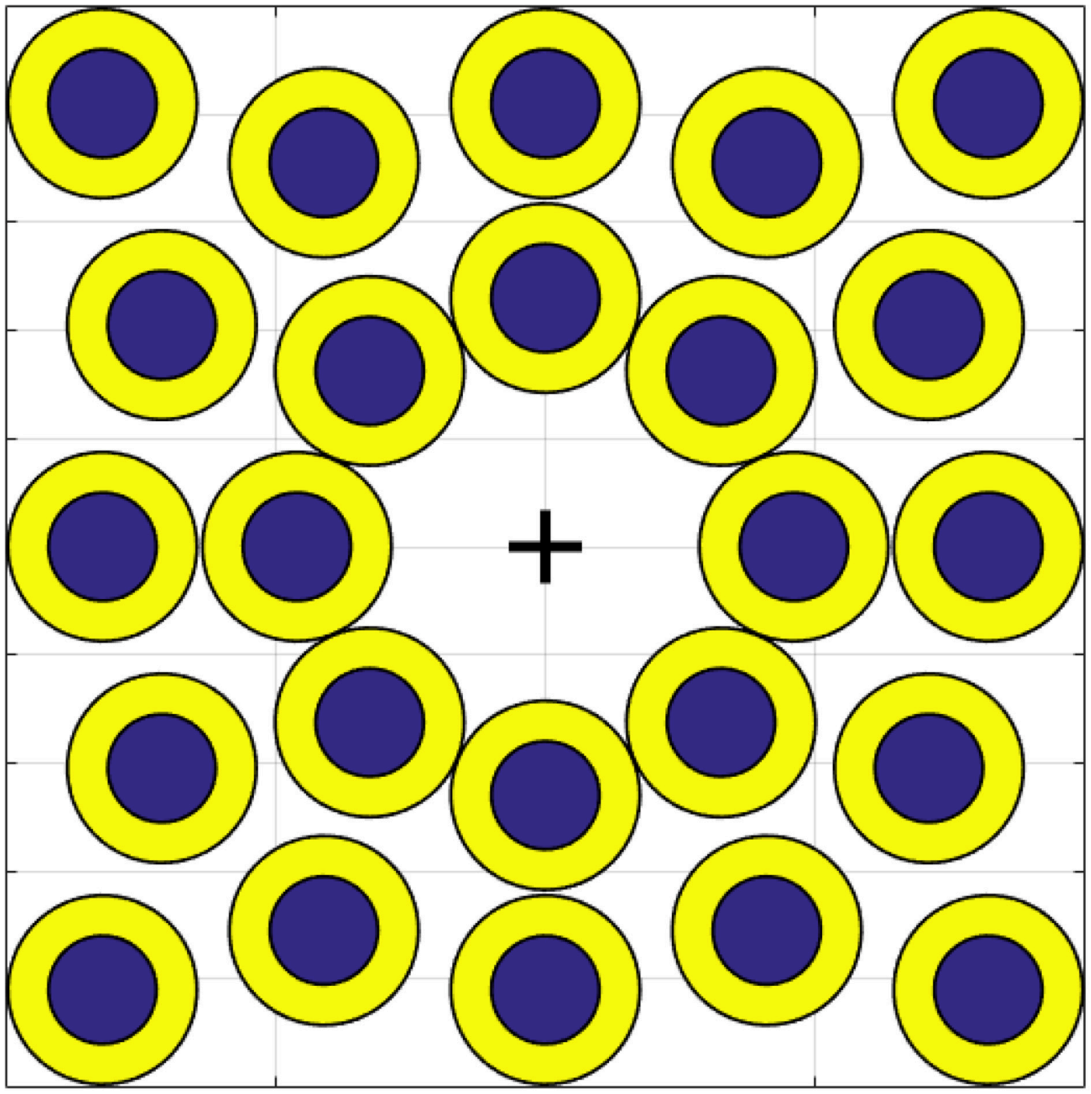
Position of each target on the virtual environment. The bigger circles, in yellow, are called easy targets. Smaller ones, in blue, are called hard targets. The cross indicates (*c*_1_, *c*_2_) = (0, 0).

For each map, subjects were instructed to acquire 48 circular targets. The starting point was always the center of the plane, i.e., (*c*_1_, *c*_2_) = (0, 0). The position of each target is reported in Figure 10. A target was considered acquired if the pointer remains inside the area of the target for 1 s, then the circle disappeared. Subjects have a time window of 20 s to try acquiring each target. After that time the target disappeared. The target changes color from red to green when the pointer is acquiring it. Each time the target disappears, subjects had to take the pointer back to the center of the plane. Only when this happens the subsequent trial can start.

As shown in Figure 10 there are two kinds of target, the biggest ones in yellow are the easiest and the smallest ones in blue are the hardest to acquire. The sequence of targets and the order of the maps were randomized for each subject. In order to make participants equally familiar with each map, the target sequence was preceded by a training phase. The experiment was split in two sessions of half an hour each in order to avoid over straining the subjects. During the first session the subject tested each map on the easy targets. During the second session, the subject had to perform the same task on the hard targets.

All the subjects were asked to complete a questionnaire after the experiment.

### 4.5. Results and Discussion

The tested maps are grouped in three sets, EMGs-only maps (EMGs), Augmented (AUG) and benchmark (BM). In accordance with (Williams and Kirsch, 2008; Scheme et al., 2014), we evaluate the performance of the maps by using the following metrics:

- Completion rate: percentage of targets acquired by a subject.
- Mean time: average time spent in acquiring each target.
- Efficiency: Minimum distance between the origin and the target divided by the distance traveled to reach the target the first time.
- Overshoots count: the number of times that the pointer tries to acquire the target but loses it.
- Stopping distance: distance traveled under the target area during acquisition.

**Figure 11:**
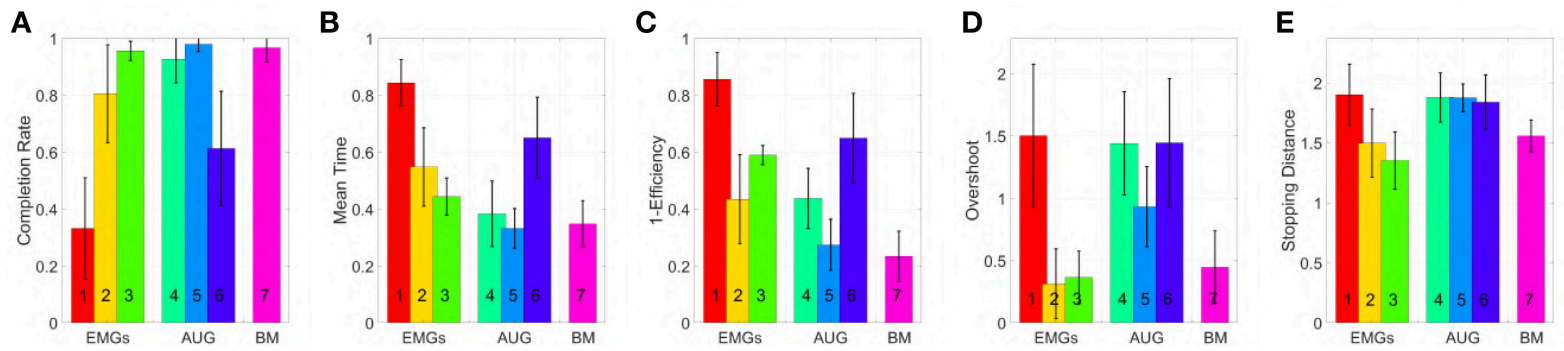
Comparison of the maps in virtual environment, evaluated through the metrics: **(A)** Normalized completion rate; **(B)** Mean time normalized to the maximum time given to complete each task; **(C)** Efficiency of the performed path compared to the minimum possible path; **(D)** Number of overshoots performed before acquiring the target; **(E)** Stopping distance traveled inside the target area. Note that, among those metrics high values are associated with good performances only in **(A)**, while the vice versa holds for **(B-E)**.

**Figure 12:**
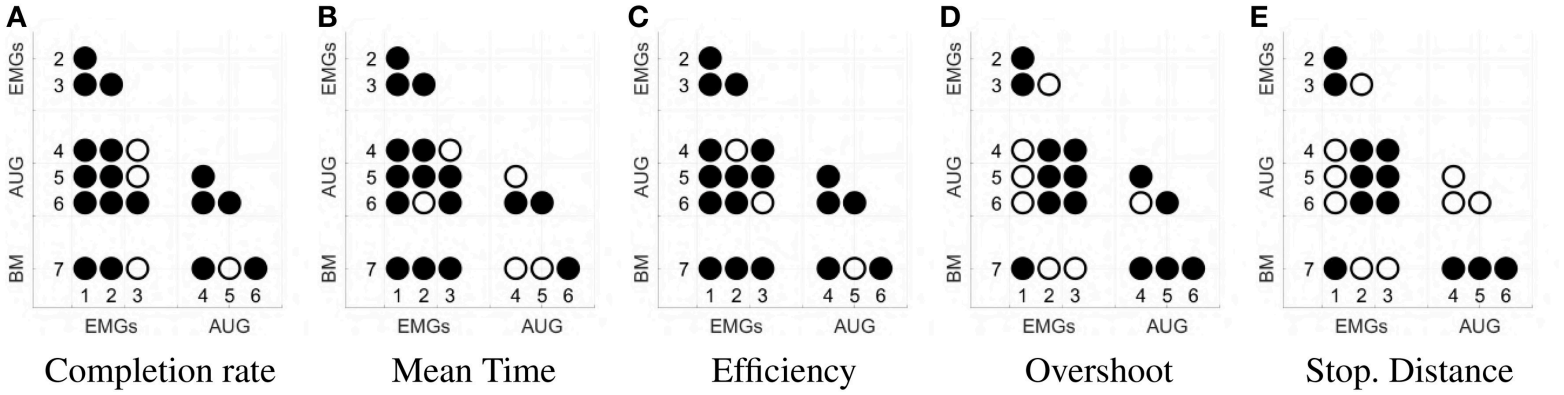
Statistical significance of each comparison between the maps. Panel **(A)** shows the results for completion rate, **(B)** for mean time, **(C)** for efficiency, **(D)** for the overshoot, **(E)** for the Stopping Distance. The significance is evaluated with a binomial test for panel **(A)**, and with a *t*-student test for panels **(B–E)**. Both analyses are done with a significance threshold of 5% and Bonferroni correction. A filled dot in the i-j-th position means that the comparison between maps i and j is statistically relevant. An empty dot indicates the contrary.

Figure 11 shows mean and standard deviation across all subjects and all targets for each index. Figure 12 shows which of the pairwise comparisons are statistically relevant for each metric presented. We verify the statistical relevance of the completion rates through a binomial test with 5% of tolerance, since the index follows a binomial distribution by construction. *T*-test with 5% of tolerance is instead used for verifying the statistical relevance of the comparisons between remaining indexes. We operate a Bonferroni correction of both the tests, for taking into account that the maps are compared multiple times. Since the tested indexes are the sample mean of a large number of trials that we can hypothesize independent and identically distributed, it is reasonable to assume their distribution to be Gaussian. However, prior to performing the *t*-test, we statistically evaluated the Gaussianity of the data. First, we used a leave-out strategy for generating samples of the sample mean by randomly picking the 80% of the total measurements. One hundred samples were generated in this way for each index. Second, we used the Kolgomorov-Smirnov test, with confidence level 5%. All the tests returned a positive outcome.

According to the Completion rate results (see Figure 11A), the first and the sixth maps are the hardest to use. Mean time (see Figure 11B) suggests that maps three and five are, on average, faster to reach each point on the virtual plane.

Efficiency (see Figure 11C) measures how convoluted is the trajectory followed to reach the target. The fifth map is the only one that achieves performance comparable to the benchmark, with the first one performing the poorest. Oveshoots counts (see Figure 11D) show that maps using EMGs only make fewer overshoots, with the exception of the first one. Finally, the Stopping distance (see Figure 11E) is similar among all the maps.

These results suggest that third and fifth maps were in general better than the others in their groups. When using these maps, subjects performed very closely to the benchmark case, even if the amount of signals required was smaller.

Subjects that participated to the experiment were also asked to complete a questionnaire. The goal of this questionnaire was to collect information about aspects difficult to measures otherwise, such as how intuitive the algorithms are, or how tired subjects are after the experiments. To evaluate each field of the questionnaire a Likert-like scale has been used (Likert, 1932). Subjects specify their level of agreement with each sentence by a value from − 3 to 3. Questions were asked for each map, including the benchmark. The sentences are listed below

1. During the trial I was perfectly isolated from external distractions.
2. I'm not tired at all.
3. Moving the pointer in the right direction with this map was very intuitive.
4. In my opinion, it was easy to maintain the pointer under the target area.
5. I think I have improved my ability during the trial.

**Figure 13:**
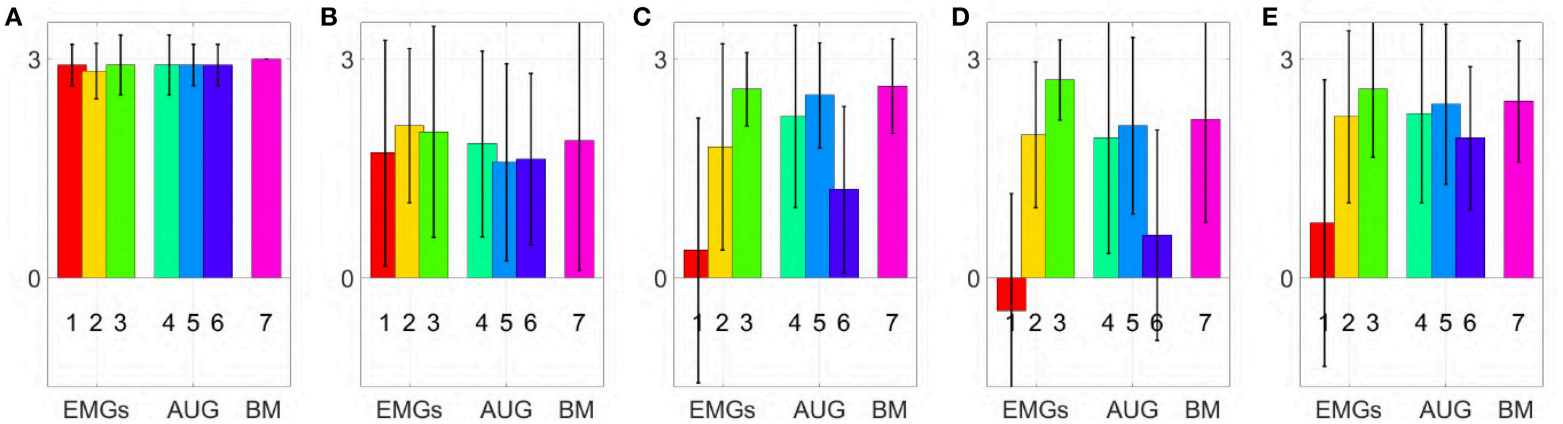
Comparison of the maps in virtual environment, evaluation of the questionnaire statements (min −3, max +3); **(A)** During the trial I was perfectly isolated from external distractions, **(B)** I'm not tired at all, **(C)** Moving the point in the right direction with this map was very intuitive. **(D)** In my opinion, it was easy to maintain the pointer under the target area. **(E)** I think I have improved my ability during the trial.

Figure 13 shows mean and standard deviation of the evaluations provided by the subjects. This questionnaire shows that subjects were well-isolated from external distractions. Furthermore, subjects were not too much tired at the end of the experiment. In general, the questionnaire confirmed that the 3rd and 5th maps are more intuitive than the others, with overall performance comparable to the benchmark. Note however that all the results comes with a considerable variance, so no definitive statement can be done in terms of general validity of the comparison.

## 5. Qualitative Experimental Validation With Able-Bodied Subjects

The following experiment aims at testing qualitatively maps 3 and 5, together with the benchmark, in controlling a soft artificial limb in real life applications. This test serves as a first validation toward prosthetic application, which will be addressed in future work.

### 5.1. Experimental Setup

**Figure 14:**
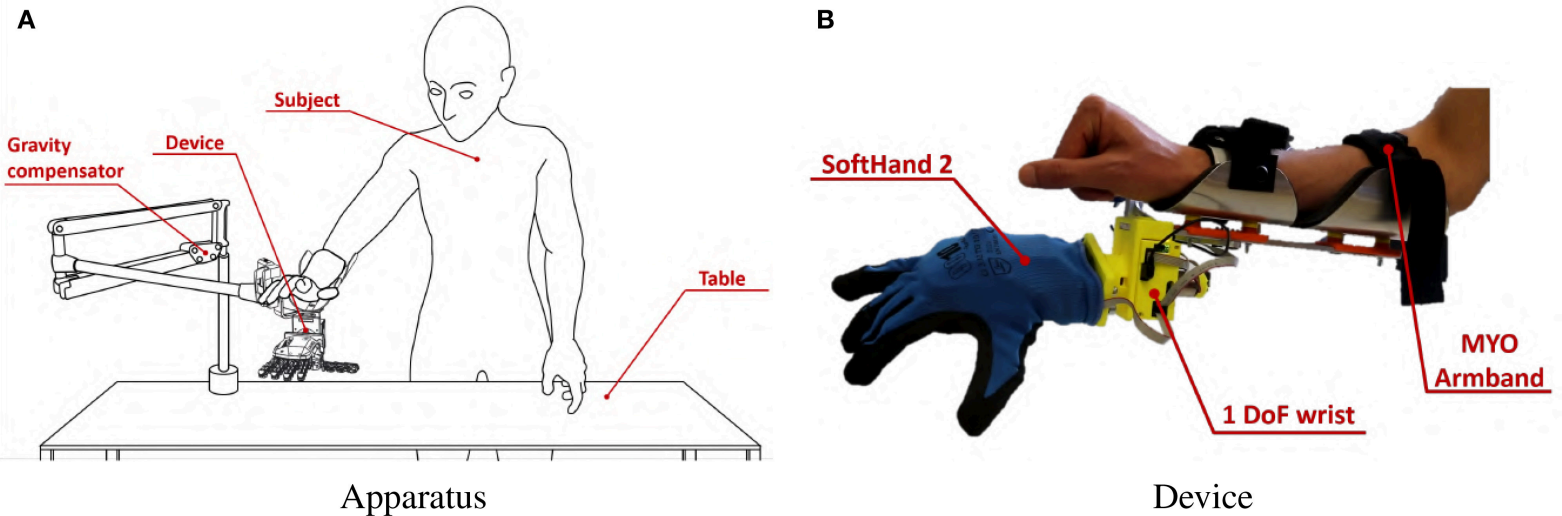
Experimental Validation apparatus. The subject, wearing MYO Armband, was instructed to stand in front of a table, as shown in panel **(A)**. She or he was then asked to wear the soft artificial limb on the right forearm near the wrist, as also shown in panel **(B)**. A gravity compensator is used to alleviate the burden of the hand prototype.

**Figure 15:**
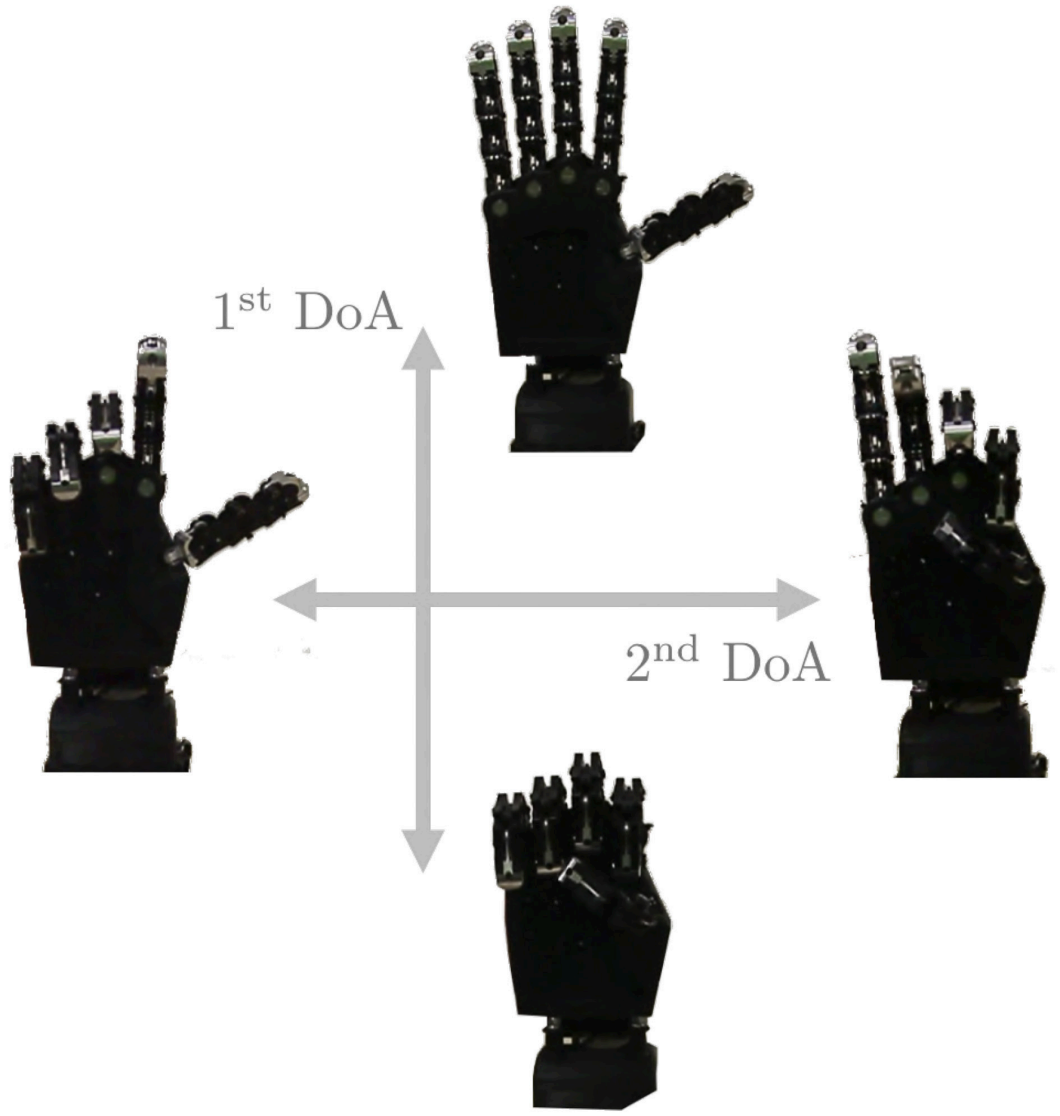
The two degrees of actuation move SoftHand 2 (Della Santina et al., 2018) along the two movements presented in figure. The first DoA is controlled by the signal *c*_1_ while, when used, the second DoA is defined by *c*_2_.

The experimental setup is shown in Figure 14, and it is composed by:

- **MYO Armband**; See section 4.2 for a description of this device.
- **Prototype**; In order to test the controllers on different types of DoA, a prototype with three motors has been used. This device consists in a soft right hand connected to an actuated wrist capable of pronation-supination motions. As soft hand we employed Pisa/IIT SoftHand 2 (Della Santina et al., 2018), an under-actuated hand with 19 degrees of freedom. It implements the two degrees of actuation shown in Figure 15, and inspired by the most common human hand postures (namely postural hand synergies) as found in (Della Santina et al., 2017a). This robotic hand has a self-contained design and it is actuated with a transmission system encompassing just one tendon, pulleys and two motors. SoftHand 2 demonstrated good grasping skills in different working conditions, combined with a high level of robustness. The active wrist is made of a MAXON DC-X 22s 12V motor and a magnetic encoder. The rotational DoF is placed axially to the length of the artificial limb, to implement the active prono-supination motion of the wrist.
  During experiments, the prototype was used in two different operating modes. Each mode uses only 2 DoAs per time:
  1. **1 DoA Hand + wrist** [OM1]: We activate the first DoA of the soft hand and the wrist. In this way the dexterity of the prototype is equally split between the wrist and the hand.
  2. **SoftHand 2** [OM2]: We activate both the DoA of SoftHand 2. The wrist is maintained fixed with the hand palm plane parallel to the subject's palm plane. In this way all the dexterity of the prototype is allocated on the hand.
- **The zero gravity SaeboMAS**: is a passive gravity compensation arm^3^ that we use to support the upper limb during the experiments. In this manner muscular fatigue is prevented during task execution. The use of a soft hand in combination with this device has been explored in (Ciullo et al., 2018).
- **Mechanical interfaces**: the custom made mechanical parts necessary to fix the artificial limb to the subject's arm, and to connect the SaeboMAS. We point the reader to Ciullo et al. (2018) for more details on this mechanical components.

The control algorithms are implemented in Matlab 2018 on a dedicated computer, while the low level control of all the devices is implemented directly onboard on the custom electronics described in Della Santina et al. (2017b).

**Figure 16:**
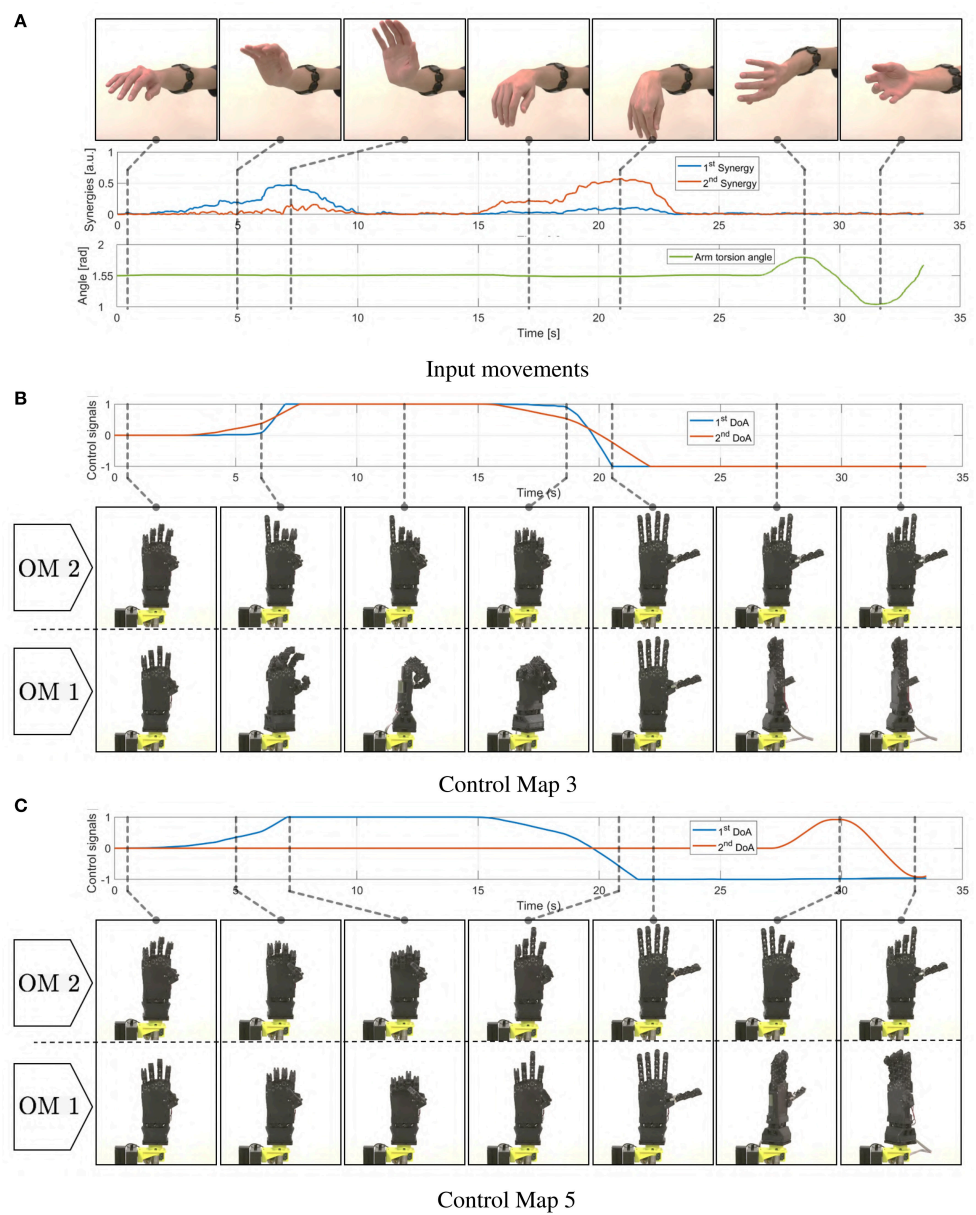
Motions induced in the soft artificial limb as a consequence of simple movements of the operator's upper limb. In panel **(A)** the operator's movements are presented, together with corresponding myoelectric (EMG) and postural measurements (IMU). In **(B)** we show the resulting *c* signals produced integrating the EMG signals through map 3. The same panel depicts the resulting motions of the device in both operating modes. Similarly panel **(C)** shows evolutions of *c* and hand postures when map 5 is used.

In Figure 16 we report the behavior of the whole system in response to a simple pattern of motions of the subject's limb. EMG and posture signals are reported, together with the corresponding outputs of the maps. The device motions are shown for both modes.

### 5.2. Participants

Eight able-bodied subjects took part in the experiment, seven men and one woman with age ranging from 23 to 32. All subjects were tested on their dominant hand (right hand; self-reported hand dominance). All participants were naive to the experimental purpose of the study and had no history of neuromuscular disorders. These subjects were not the same ones who participated in the Virtual Environment Validation experiment. Before data collection, subjects signed an informed consent to participate in the experiment. The experimental protocols were approved by the Institutional Review Board of University of Pisa, in accordance to the Declaration of Helsinki.

### 5.3. Experiment

During this experiment we tested the 3rd and the 5th maps, together with the benchmark. Each map was tested on each operating mode of the soft prototype described in subsection 5.1. Subjects, still wearing the MYO Armband from the calibration phase, were instructed to stand in front of a table. They were asked to wear the artificial limb on the right forearm near the wrist. A representation of the entire apparatus is reported in Figure 14.

The six combinations of the three maps and the two operating modes were tested on the following tasks:

- **Box and Blocks:** This is a standard test widely used to evaluate the unilateral gross manual dexterity of post-stroke patients or upper limb amputees using prosthesis (Desrosiers et al., 1994). During this task a rectangular container is placed in front of the subjects. It is divided into two square compartments of equal dimension. Some colored wooden cubes are placed in one compartment. The subjects are instructed to move as many blocks as possible from one compartment to the other for a period of 60 s.
- **Pyramid:** This task aims at evaluating the precision manual dexterity needed to move little objects. During this test participants have to build a pyramid with the same wooden blocks as those used in the previous task. Five cubes are placed in front of the subject as to form the base of the pyramid. Other two groups of five blocks are placed on the left of the subject. During the task subjects take blocks from these two groups and place them onto the pre-built pyramid base on the right. Sixty seconds are available to accomplish the task. The pyramid's size was designed so that is particularly challenging to complete it in the given time.
- **Cards:** During this task three cards are placed on the table in front of the subject. The participants are instructed to turn upside down each card, one at a time, for a period of 60 s. If the subject turns upside down all three cards before the time is up, then, she or he is instructed to repeat the task. This task is inspired by that included in the Jebsen Hand Function Test (Dromerick et al., 2008).

An evaluation scale has been introduced, to evaluate the performance obtained by each subject during this experiment. Subjects were informed about this scale before the experiment started. For each task, points were assigned as described below:

- **Box and Blocks:** One point for one or more cubes moved into the empty compartment at a time.
- **Pyramid:** One point for each cube correctly positioned.
- **Cards:** One point for each card correctly turned upside down.

Each task was repeated twice to obtain a more consistent dataset. The sequence of maps was randomized for each subject. To avoid straining the subjects, each one tested only one of the two prototype operating modes. To familiarize participants with each test, the tasks were preceded by a 2-min training phase in which the operators were left free to experiment with the device and the map. Furthermore, subjects were helped to understand tasks and maps with videos, showing the prototype behavior for each combination of operating mode and control map. These videos are available as multimedia attachment to the present paper.

Subjects were asked to complete a questionnaire after each the experiment. Questions were asked for each map, including the benchmark. To evaluate each field of the questionnaire a Likert-like scale was used. Hence, subjects specified their level of agreement with each sentence by a value from -3 to 3. The fields of the questionnaire were:

1. I was able to move the artificial hand easily in the right way.
2. During the trial I was perfectly isolated from external distractions.
3. I think this is the best controller among all others.

### 5.4. Results and Discussion

**Figure 17:**
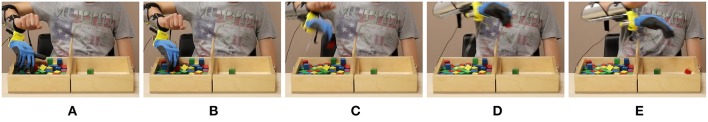
Photo-sequences of a single block transport in the Box and Blocks task. Map 3 (EMG-only) is used, and the device is in mode 2. In panels **(A,B)**, the operator exploits both DoAs of the soft hand to pick the red block through a pinch grasp. In panels **(C,D)** the block is firmly maintained between thumb and index while being moved from let to right. In panel **(E)** the operator opens the hand, and releases the block into the empty box.

**Figure 18:**
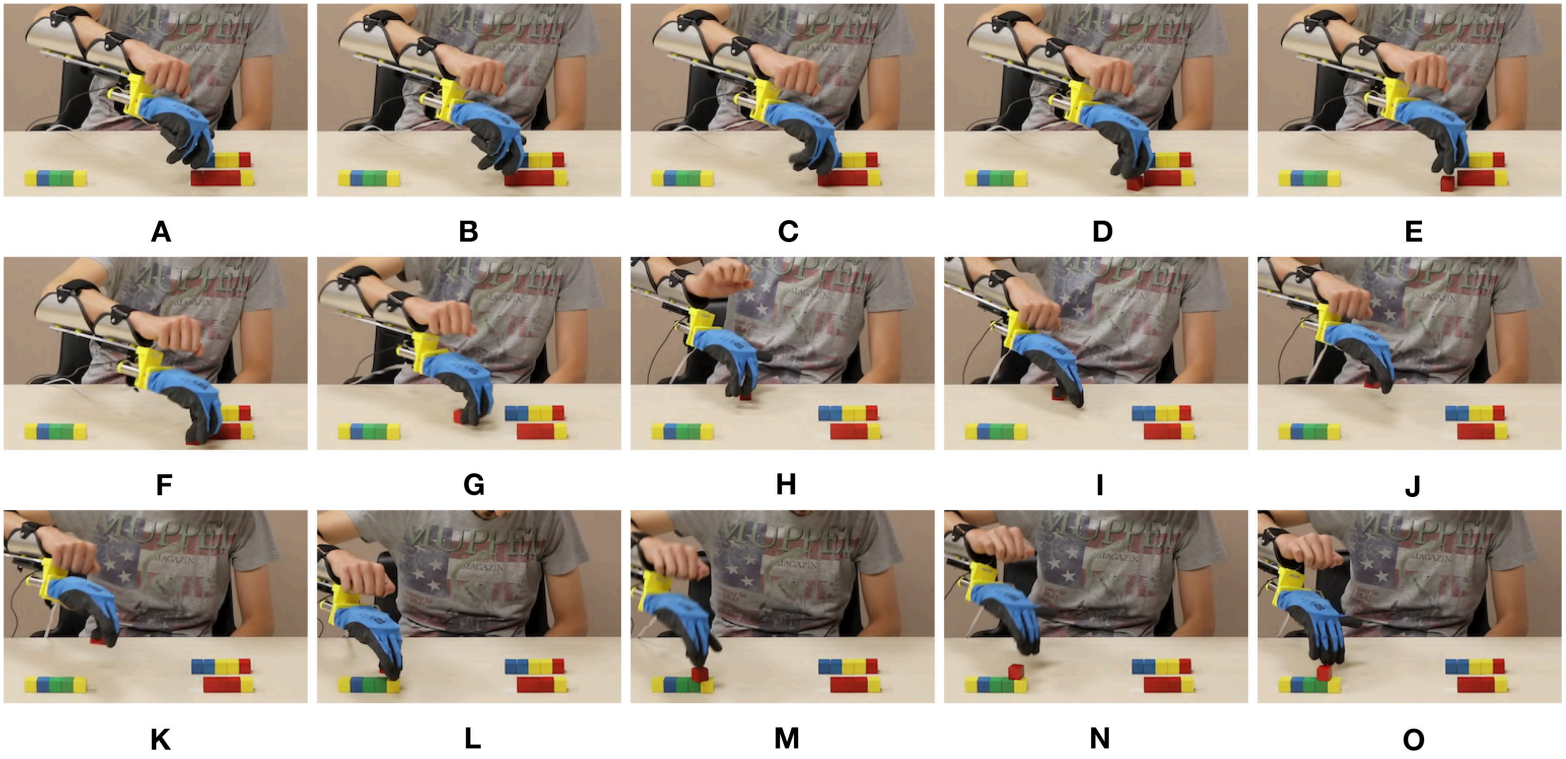
Photo-sequences of a single block placement in the Pyramid task. Map 3 (EMG-only) is used, and the device is in mode 2. In panels **(A–E)** the operator separates the block from the group by extending the index finger. In panels **(F–H)** the block is pushed toward a position more suitable for performing the pinch grasp which happens in panels **(I,J)**. In panels **(K–M)** the hand is opened and the cube left on the pyramid base. Finally in panels **(N,O)** the block is pushed in the correct place through the extended fingers.

**Figure 19:**
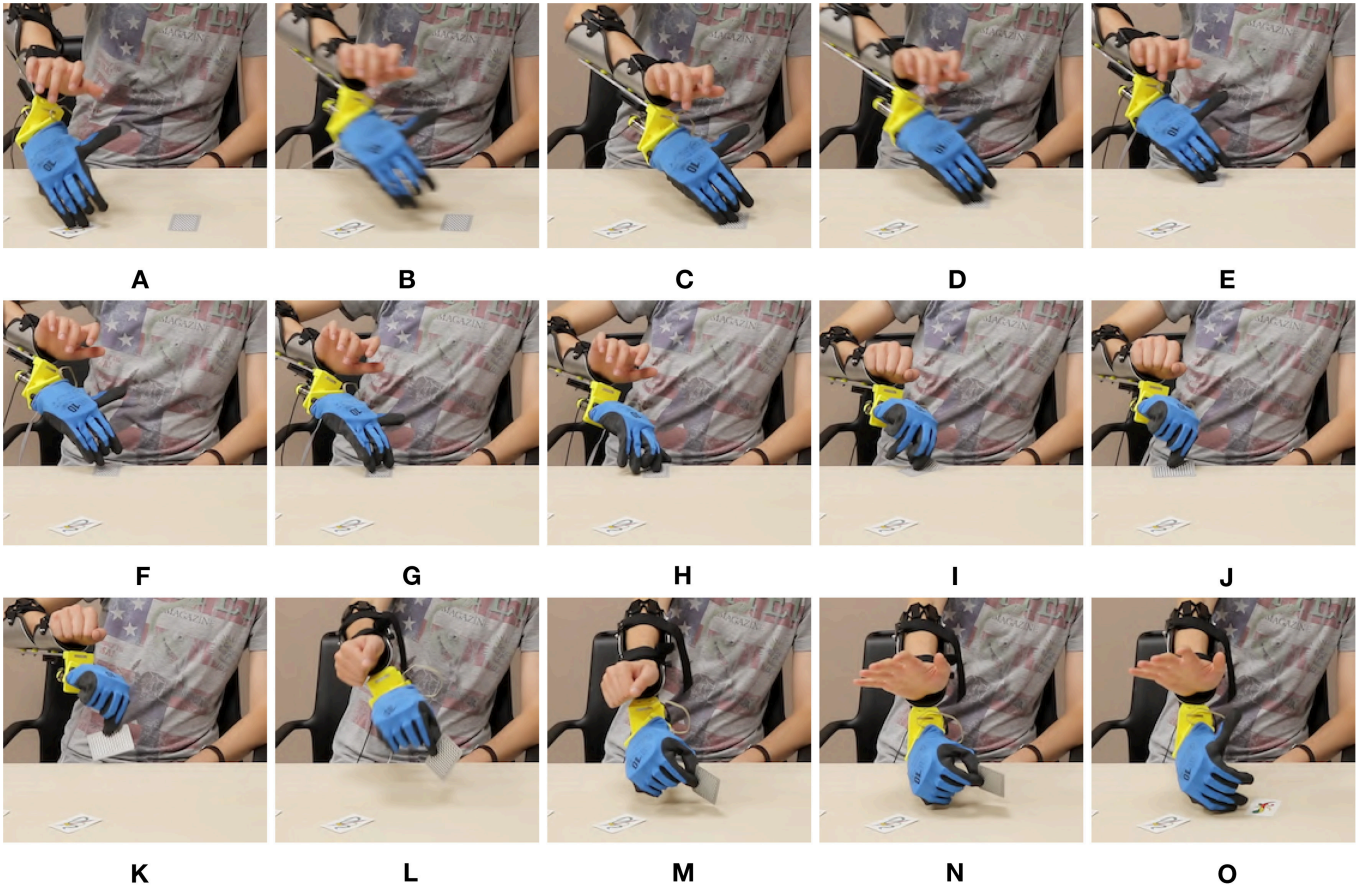
Photo-sequences of a single card turning in the Card task. Map 5 is used, and the device is in mode 1. In panels **(A–C)** the soft hand moves from the previously turned card to the new card, which is moved to the edge in panels **(D-F)** by sliding it on the table. The wrist posture is planned so as to facilitate the sliding. In Panels **(H–K)** the card is picked between thumb and index. In panels **(L–O)** the operator flips the card by rotating the wrist.

In Figures 17–19 we present extracts of an expert operator performing the three considered tasks. In performing these tasks the subject makes large use of the second DoA of the Pisa/IIT SoftHand, and of the wrist's motions. Note that these degrees can be controlled with only two independent electromyographic signals thanks to the proposed maps. In Figure 17 a block is picked between index finger and moved from/to the empty box. To perform the pinch grasp the operator had to exploit the second degree of actuation of SoftHand 2. In Figure 18 the operator picks a block from one of the groups on his left, and places it over the first level of the pyramid. The second DoA of SoftHand 2 is used first to move the block from its group by extending the index finger, then to pick the object between index and thumb fingers. In Figure 19, the wrist is extensively exploited for positioning the hand in postures advantageous for the operations. In panels (A–E) the orientation is such that the card is well-visible to the user. In panels (F–J) the orientation is changed so as to enable the execution of an effective grasp. Finally in (K–O) the wrist is used for actively rotating the cart.

**Figure 20:**
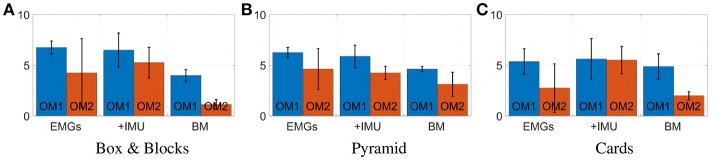
Photo-sequence of an activity of daily living performed using the device in mode 1, with Map 5. The operator rotates the wrist so as to have the soft hand in a good posture to perform a grasp, in panels **(A-D)**. The power grasp is established in **(E,F)**, and firmly maintained while the cap is removed with the free hand. Motions of the artificial wrist are exploited to pour water from the bottle into a glass in panels **(G-N)**. In **(O)** the grasp in maintained while the cap is plugged again.

Figure 20 displays mean value and standard deviation of the collected data for each task for all subjects. Most subjects were able to manage the artificial limb in both operating modes with all three considered maps. Maps 3 and 5 have similar performance in the operative mode 1 (OM1), while map 3 slightly outperforms map 5 in the operative mode 2 (OM2). Interestingly both proposed maps outperform the benchmark in both operative modes, despite the fact that the benchmark is using double the amount of EMG signals to control the same amount of degrees of actuation. Of course these results should not be considered as definitive; different associations between *s* and *c*_p_, and more tasks should be considered to that end.

However these experiments suggest that using less signals in a more clever way can actually generate performance comparable to using a larger number of them. Furthermore, when it comes to add information it seems convenient to introduce postural information instead of further sEMG.

**Figure 21:**
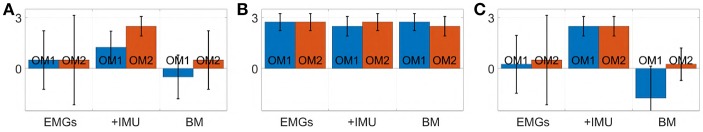
Photo-sequence of an activity of daily living performed using the device in mode 2, with Map 3. The operator picks the red candy among the white ones, by performing a precision grasp in panels **(A–C)**. In **(D,E)** the candy is lifted. In **(F–I)** the grasp is firmly maintained while the candy is unwrapped with the free hand. Finally, in **(J)** the operator eats the candy. The operator signed a written informed consent for the publication of this image.

These conclusions are also supported by the results of the questionnaire. Figure 21 shows mean and standard deviation of the evaluations. According to panel (A), subjects considered more natural the use of map 5 in both operative modes. This is also reflected in an overall higher appreciation of the map, as proven by panel (C). Panel (B) confirms that subjects were well-isolated from external distractions. Furthermore, the questionnaire suggests that subjects preferred the map combining IMU and EMGs.

**Figure 22:**
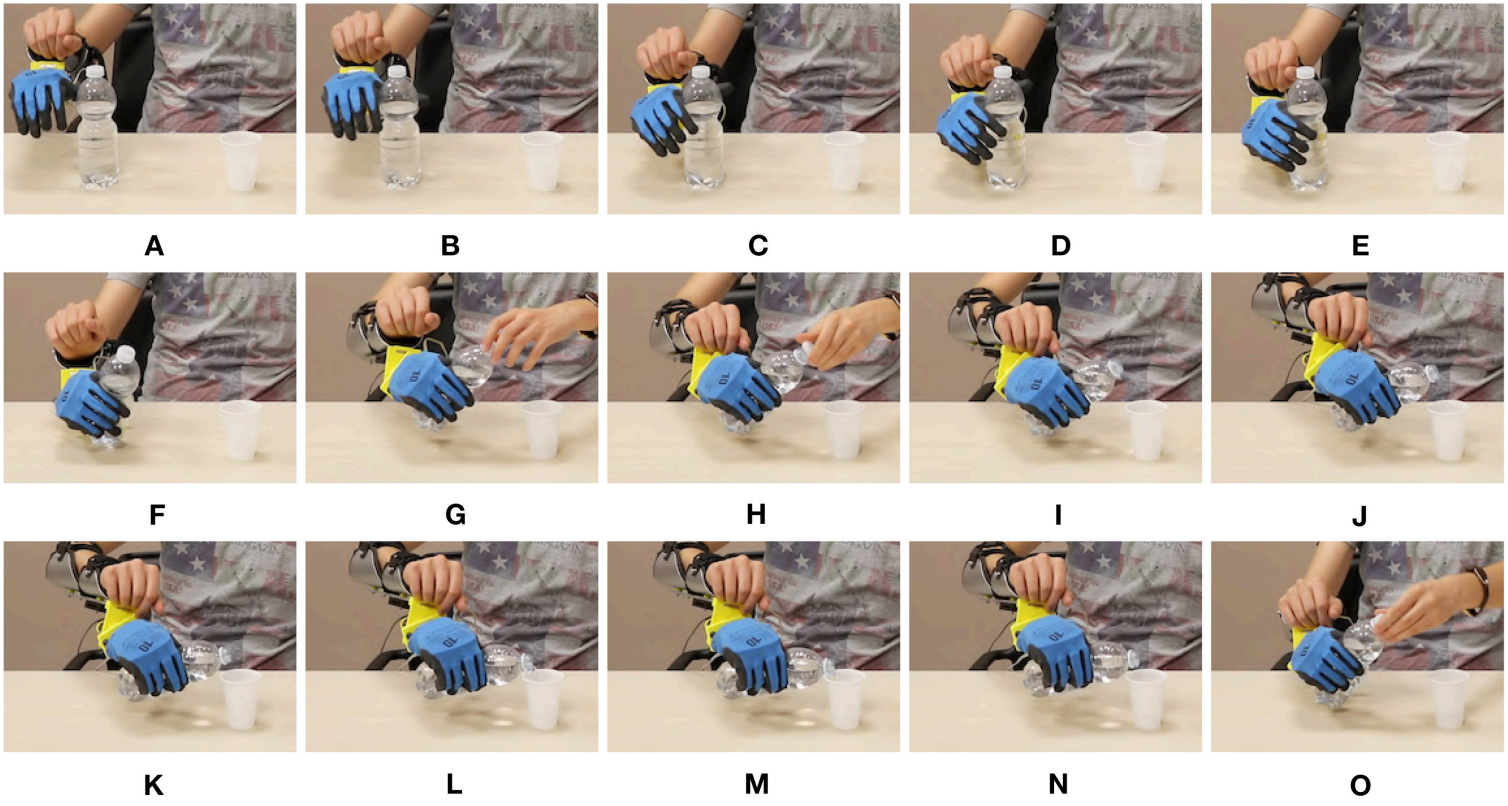
Mean value and standard deviation of points scored by subjects during each task. Blue bars represent the prototype's first operating mode, OM1. Red bars represent the prototype's second operating mode, OM2.

**Figure 23:**
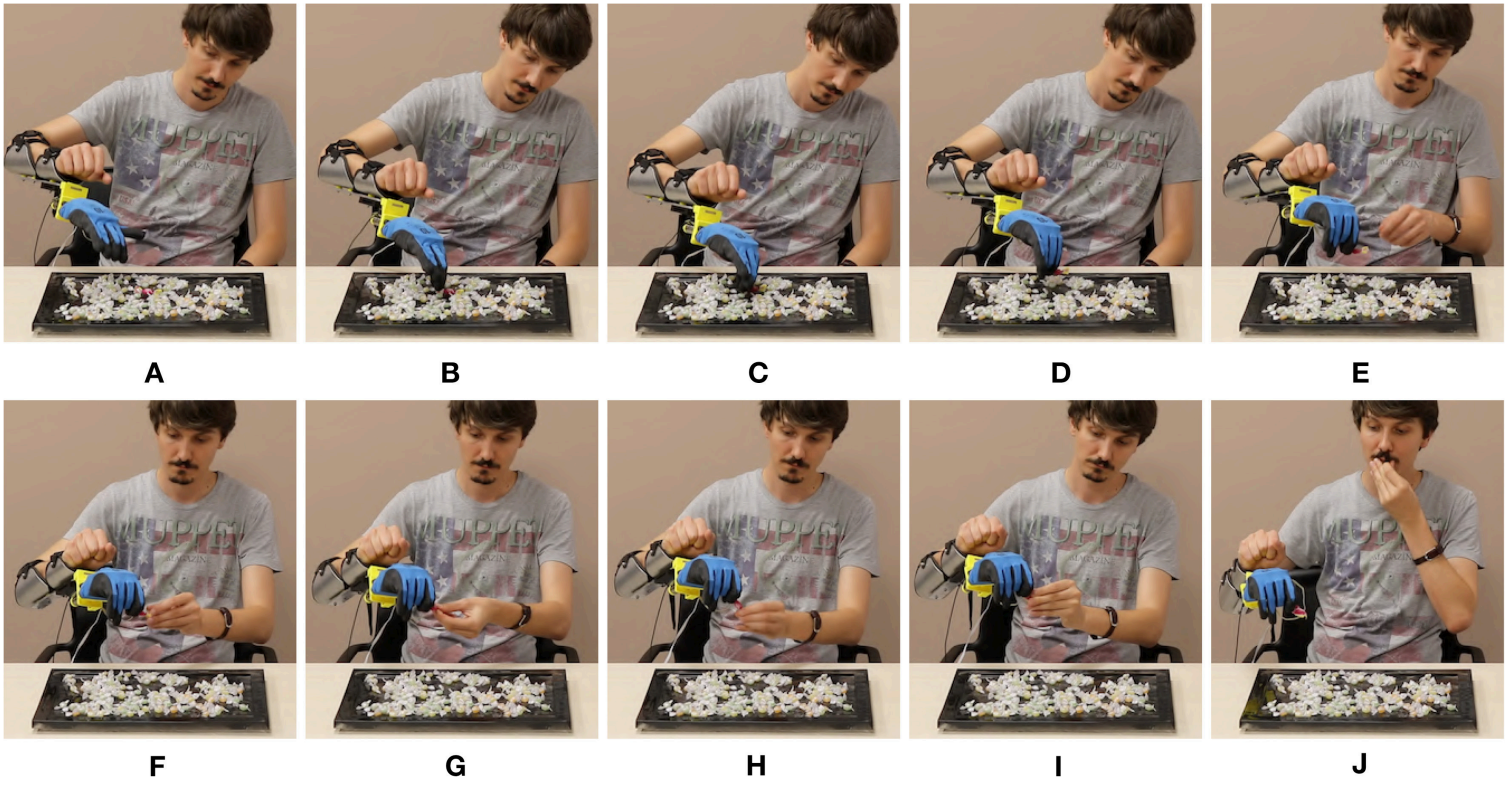
Evaluation of each statement of the questionnaire (min −3, max +3). Panel **(A)** reports the evaluation for “I was able to move the artificial hand easily in the right way.” Panel **(B)** reports the evaluation for “During the trial I was perfectly isolated from external distractions.” Panel **(C)** reports the evaluation for “I think this is the best controller among all others.” Blue bars correspond to the prototype's first operating mode, OM1. Red bars correspond to the prototype's second operating mode, OM2.

In Figures 22, 23 we show two other examples of daily living activities performed by the expert user using the soft artificial limb. In Figure 22 the device is in operating mode one, and controlled through map 5. The operator grasps a bottle and pours some water into the glass through wrist motions. In Figure 23 the device is in operating mode two, and controlled through map 3. The operator picks a candy using a pinch grasp. The candy is stably held while it is unwrapped.

## 6. Exploratory Experiments on Subjects With Limb Loss

We conclude the study by testing the proposed strategies with two subjects with limb loss. The first subject (37, female) is an user of cosmetic prostheses, and an occasional user of myoelectric prostheses. She has a congenital malformation at the trans-radial level in the left hand. The second subject (41, male) is an user of body powered and myoelectric prostheses. He was amputated 8 years ago, at the trans-radial level in the left hand. Therefore, both of them could have a different disposition of the muscles, and they could not be used or able to activate the muscles to execute activities of daily living.

Neither of the two subjects could test the benchmark, since both of them had only two independent EMG signals to provide. With the first subject the reason was physiological. Indeed—as already discussed in the introduction and shown by Figure 2—she was able to generate only two independent signals with her residual stump. With the second subject the reason was technological. His socket—on which we plugged the SoftHand2—is a standard setup that includes two Ottobock surface EMG sensors. Both reasons are very common, and represent well the kind of situations that we can encounter in practice.

**Figure 24:**
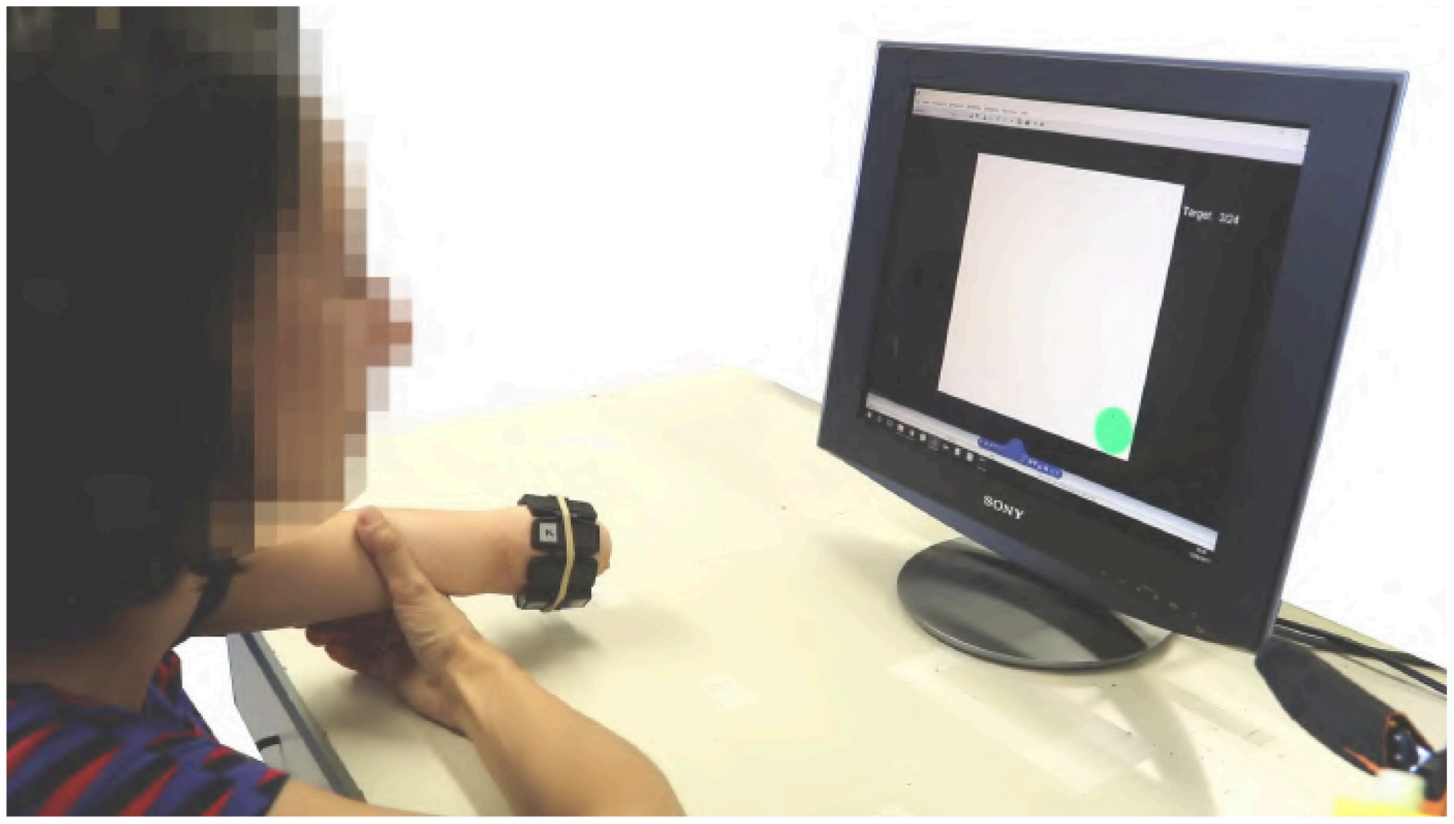
The first Subject while performing the experiments in the virtual environment. The MYO Armband is placed on the setup. Both Map 3 and 5 are tested. The benchmark could not be tested because it was not possible to extract enough independent signals from the subject.

**Figure 25:**
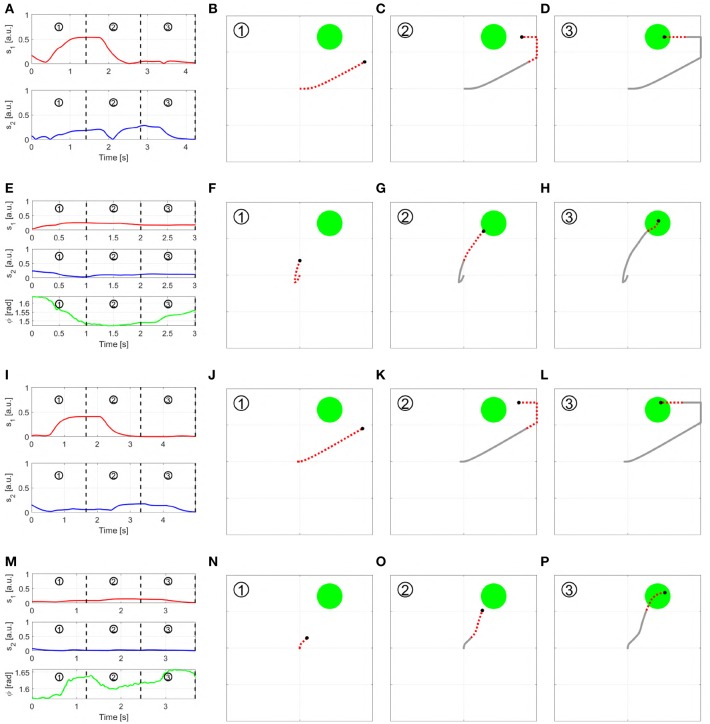
Outcome of a trial in virtual environment, performed by a able bodied subject **(A–H)** and the first subject with limb loss **(I–P)**, using both Map 3 and 5. More specifically: **(A–D)** show the able-bodied subject using Map 3 and **(E–H)** using Map 5; **(I–L)** show the subject with limb loss using Map 3 and (m-p) using Map 5. Each row reports the input signals performed to reach the target (first column), and the path of the pointer split into three sequential parts. The red and dashed part of the line show the motion produced in the time segment, while the gray solid line show the past trajectory.

**Figure 26:**
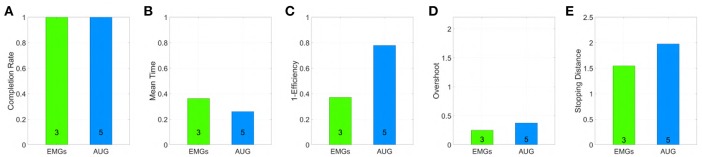
Performance obtained by an amputee using maps 3 and 5 in virtual environment, evaluated through the metrics: **(A)** Normalized completion rate; **(B)** Mean time normalized to the maximum time given to complete each task; **(C)** Efficiency of the performed path compared to the minimum possible path; **(D)** Number of overshoots performed before acquiring the target; **(E)** Stopping distance traveled inside the target area. Note that, among those metrics high values are associated with good performances only in **(A)**, while the vice versa holds for **(B–E)**.

The first subject performed experiments in the virtual environment, according to the full protocol described in section 4. Figure 24 shows the subject performing the experiment. She achieved 100% success rate with both maps. Figure 25 compares the behavior of an able-bodied subject and of the first subject in acquiring the same target, with both maps. Figure 26 reports the performance of the subject according to the metrics introduced in section 4.

**Figure 27:**
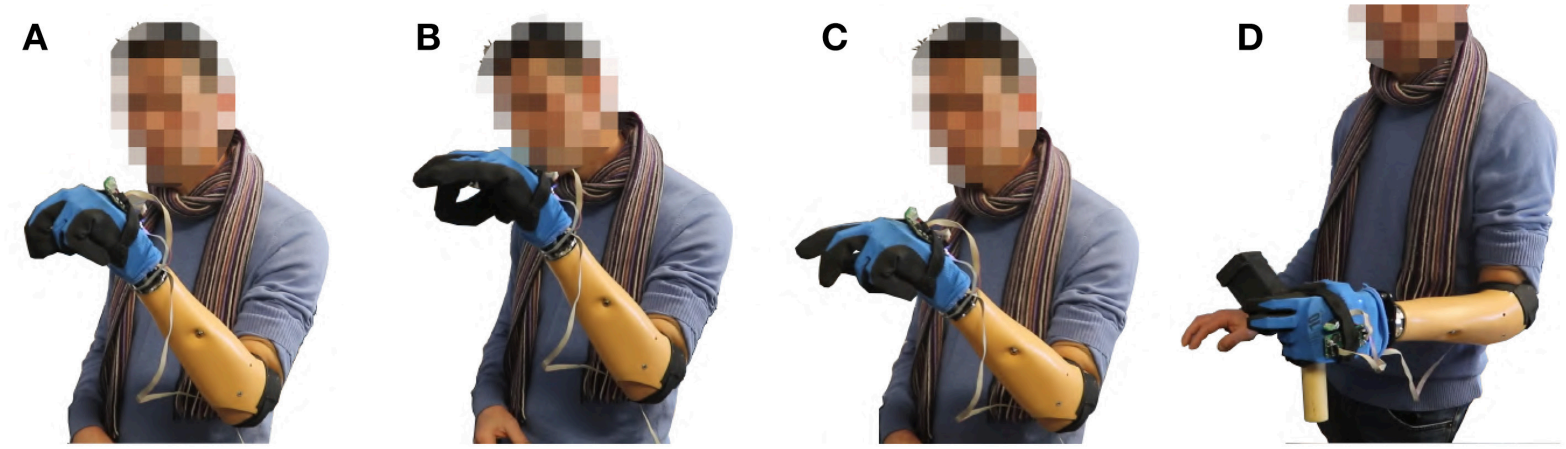
The second Subject controls the SoftHand2 using map 3. No wrist is considered here. In panel **(A)** the prosthetic hand assumes a posture coherent with a power grasp. In panel **(B)** the prosthetic hand assumes a posture coherent with a pinch grasp. In panel **(C)** the index of the finger is extended, as for pushing a button. In panel **(C)** two different objects originally distant between each other are grasped together—see Video 1 for more details.

**Figure 28:**
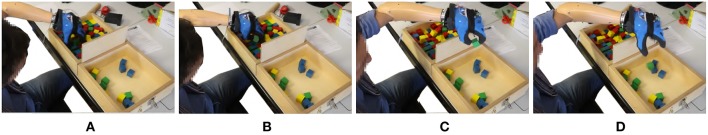
The second Subject perform Box and Blocks task, using Softhand2 controlled with map 3. No wrist is available here. The ability of the hand to perform pinch grasps is exploited to grasp the block with good precision. The final score achieved is 10 cubes moved in 1 min.

The second subject performed instead experiments with the physical prototype. We removed here the active wrist, and we connected the SoftHand2 directly to his socket. The connection happened through a mechanical interface compatible with Ottobock wrist, that we had to realize *ad hoc*. No IMU was available on board, although it will be possible to easily add it in future work. So only Map 3 with OM 2 could be tested. Figure 27 shows the subject moving the hand to several meaningful postures—panels (A–C)—and grasping two objects at the same time that were originally far from each other—panel (D). Figure 28 shows a photosequence of the subject performing Box and Blocks tasks. The protocol followed was the same discussed in section 5.1. His score on two trials has been of 10 blocks in 1 min. We point the interested reader to the multimedia attachment, which includes the video of both these and further experiments.

While very preliminary, these results prove that the proposed algorithms can make it possible for a subject with limb loss to effectively control prostheses with a reduced amount of independent EMG features.

## 7. Conclusions

In this work, we investigated two novel approaches for proportionally and simultaneously controlling an artificial limb, with a reduced amount of independent EMG signals w.r.t. classic approaches. This is a compelling problem in the state of the art of prosthetics, since typically a low number of independent EMG signals can be extracted from a forearm of a transradial amputee subject. The first approach consists in combining independent signals extracted from an array of EMG readings, to obtain a same number of control signals. The second approach enriches EMG through postural information. For each approach we proposed three control maps. We quantitatively tested the performance of the maps against a state of the art benchmark, through a virtual environment we designed. The study involved twelve able-bodied subjects. Two maps having achieved the higher scores in these experiments were selected—one for each class of algorithms—and qualitatively tested in controlling a soft artificial limb. Eight able-bodied subjects took part in this second experiment. Finally, the same two maps were tested on two subjects with limb loss. The outcomes of this experimentation are promising. Our results suggest that the proposed maps can enable the control of multi-DoA artificial limbs with a reduced amount of control signals if compared to the standard approaches. Future work will be devoted to further validating the application of these algorithms to the prosthetic field, and also their potential application in the emerging field of extra-limbs.

## Ethics Statement

Before data collection, subjects signed an informed consent to participate in the experiment. The experimental protocols were approved by the Institutional Review Board of University of Pisa, in accordance to the Declaration of Helsinki.

## Author Contributions

All the authors designed the study. MM, CD, and CP performed the experiments. MM, CD, and GG performed the data analysis. MM, CP, and MC developed the experimental setup. All authors contributed to writing the manuscript.

### Conflict of Interest Statement

The authors declare that the research was conducted in the absence of any commercial or financial relationships that could be construed as a potential conflict of interest.

## Figures and Tables

**Figure A1:**
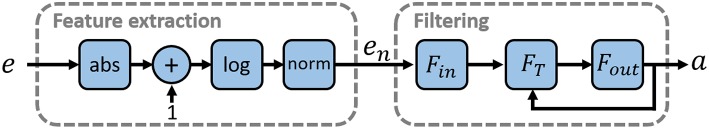
EMGs Pre-processing phase, extracting informative, and independent features from EMG acquisitions. We first perform the feature extraction, and after we filter the data using an adaptive filter.

## Footnotes

^1^This coactivation is sometimes used to control the device impedance (Ajoudani et al., 2013), but we will not consider this use further in this work.

^2^MYO armband official website, https://support.getmyo.com/hc/en-us/articles/203398347-Getting-started-with-your-Myo-armband, accessed 8-June-2018

^3^SaeboMAS official website, https://www.saebo.com/saebomas/, accessed 8-June-2018.

## Supplementary Material

Video 1 This video reports the experimental results of the application of the proposed maps in controlling the device, in both operative modes.

## A. Appendix

### A.1. Calibration

A calibration phase is executed each time a new subject performed the study, prior to the experiment. During this phase, the subject is asked to perform a sequence of flexion-extension and adduction-abduction wrist movements, following a virtual cursor on the screen. These motions are used to calculate the matrix of independent signals as described later in this section.

### A.2. Control Architecture

To test the proposed maps we implemented an instance of the general architecture introduced Section 2.1. Furthermore, as depicted by Figure 5, also pre-processing of postural information was carried out. All the algorithms are implemented by MatLab2017b scripts, and Simulink schemes.

#### A.2.1. EMG Pre-processing

Each sEMG sensor provides a zero-mean signal, the variance of which proportionally increases as a function of muscle activation. Therefore, the EMG signals were rectified to obtain a better representation of the muscular activation. In fact, as described in Ward et al. (2013), rectified EMG signals are a better predictor of the frequency components of motor unit synchronization than the raw ones.

The logarithm of the signal is then computed to compensate for the non-linear nature of the relation between EMG signals and muscular activation, as suggested in Hahne et al. (2014). Finally, a normalization is applied, yielding to

(A1) $$\begin{array}{r} e_{\text{n}}=\frac{\log\left(|e|+1\right)}{\log e_{\text{max}}}. \end{array}$$

After extrapolating *e*_n_ from the signal, a filtering is applied to reduce noise. With the aim of not introducing too much delay, we implemented a dynamic thresholding action (*F*_T_) arranged between two filters (*F*_in_, *F*_out_), as shown in Figure A1. Both are digital low-pass filters implemented as a weighted sum between the input and the output of the filter at the previous step *y*_*k*_ = α*x*_*k*_ + (1−α)*y*_*k*−1_. We choose α = 1/50. The sampling rate is 0.5 KHz. The dynamic threshold action *F*_T_ aims at neglecting small variations when the activation is almost constant. This action works as follows

(A2) $$y_{k}=\{\begin{array}{cc} u_{k}-w, & \text{if}\gamma >1\\ u_{k}+w, & \text{if}\gamma <1\\ y_{k-1}, & \text{oth.} \end{array},\text{ where }\gamma =\frac{u_{k}-y_{k-1}}{w},\text{ and }w=\beta e_{\text{f}}$$

where *u*_*k*_ is the input and *y*_*k*_ the output at the *k*−th time step. The output remains constant if the difference between input and output at the previous step is smaller than a threshold *w*. This threshold changes dynamically proportionally to the *F*_out_ filter output. β is a normalization constant equal to 0.25. The dead zone effect becomes wider proportionally to the output signal *e*_*f*_. In this way, it is possible to obtain both fast reaction when the signal is low and high oscillation attenuation for stronger activation.

#### A.2.2. IMU Pre-processing

MYO armbrand provides its posture as measured by the on-board IMU, expressed in quaternions *q* = [*q*_0_ *q*_1_ *q*_2_ *q*_3_]. In this work we use only the information of the torsion angle of the forearm ϕ. To obtain this angle from a quaternion the following transformation is needed

(A3) $$\begin{array}{r} \phi =\text{atan}2\left(2\left(q_{2}q_{3}+q_{0}q_{1}\right),\left(q_{0}^{2}-q_{1}^{2}-q_{2}^{2}+q_{3}^{2}\right)\right). \end{array}$$

No further filtering is applied to ϕ.

#### A.2.3. Independent Signal Extraction

This layer extracts the set of independent features *s* that will be fed into the control algorithms, from the filtered and rectified activations *e*. We use a procedure inspired by Choi and Kim (2011), which can be divided in two sub-phases; Factorization and Linear Regression.

**Factorization:** Consider a matrix *U* ∈ ℝ^*k*×*T*^ collecting EMG data, where *k* is the number of myoelectric channels and *T* is the amount of temporal samples. Consider also a matrix *M* ∈ ℝ^*m*×*T*^ containing the activation movements, where *m* represents the number of movement to be mapped; then it is possible to obtain the muscles synergies matrix *S* ∈ ℝ^*m*×*c*^, such that *M* ≈ *SU*. The evaluation of *S* is an optimization problem, with cost function ‖*M* − *SU*‖_2_.

**Linear Regression:** We build a linear regression for each independent signal obtained by the previous step. The matrix *B* ∈ ℝ^*m*×*m*^ is obtained by solving the equation *B* = (*SU*^*U*^T^*S*^T^)−1^*SUM*^T^. The final features are thus evaluated as *s* = *B*^T^*Sa*.

#### A.2.4. Post-processing

The last block in Figure 5 is used to manipulate the output signals from the Control Map block in order to obtain the reference signals for the output device. This means normalizing the output to the maximum excursion of the motors, adding bias to the signals in order to modify the starting point of the system and other technical operations depending on the utilized device. The two output signals are directly mapped to the two DoAs of the soft prosthesis.
